# *Alternaria* diseases on potato and tomato

**DOI:** 10.1111/mpp.13435

**Published:** 2024-03-13

**Authors:** Tamara Schmey, Christopher S. Tominello‐Ramirez, Carolin Brune, Remco Stam

**Affiliations:** ^1^ TUM School of Life Science Weihenstephan Technical University of Munich Freising Germany; ^2^ Department of Phytopathology and Crop Protection, Institute of Phytopathology Christian Albrechts University Kiel Germany

**Keywords:** *Alternaria*, defence, early blight, host‐specific toxins, metabolites, *Solanaceae*, taxonomy

## Abstract

*Alternaria* spp. cause different diseases in potato and tomato crops. Early blight caused by *Alternaria solani* and brown spot caused by *Alternaria alternata* are most common, but the disease complex is far more diverse. We first provide an overview of the *Alternaria* species infecting the two host plants to alleviate some of the confusion that arises from the taxonomic rearrangements in this fungal genus. Highlighting the diversity of *Alternaria* fungi on both solanaceous hosts, we review studies investigating the genetic diversity and genomes, before we present recent advances from studies elucidating host–pathogen interactions and fungicide resistances.

**Taxonomy:**

Kingdom Fungi, Phylum Ascomycota, Class Dothideomycetes, Order Pleosporales, Family Pleosporaceae, Genus *Alternaria.*

**Biology and host range:**

*Alternaria* spp. adopt diverse lifestyles. We specifically review *Alternaria* spp. that cause disease in the two solanaceous crops potato (*Solanum tuberosum*) and tomato (*Solanum lycopersicum*). They are necrotrophic pathogens with no known sexual stage, despite some signatures of recombination.

**Disease symptoms:**

Symptoms of the early blight/brown spot disease complex include foliar lesions that first present as brown spots, depending on the species with characteristic concentric rings, which eventually lead to severe defoliation and considerable yield loss.

**Control:**

Good field hygiene can keep the disease pressure low. Some potato and tomato cultivars show differences in susceptibility, but there are no fully resistant varieties known. Therefore, the main control mechanism is treatment with fungicides.

## Graphical abstract

We review the major *Alternaria* species that cause early blight disease complex (genetic diversity, genome structure and mechanisms of pathogenicity), management strategies such as resistance breeding and fungicide resistance.

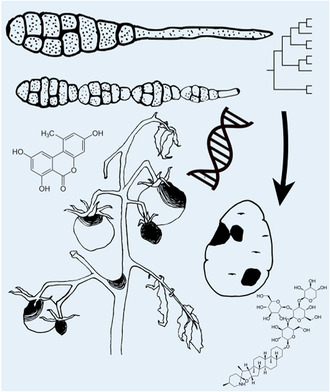

## 1 INTRODUCTION

Filamentous fungi of the genus *Alternaria* are distributed globally. Most *Alternaria* species are not only cosmopolitan but ubiquitous in natural and human‐dominated ecosystems (e.g., Lawrence et al., 2015). In a global study of soilborne fungi, *Alternaria* was the most abundant plant pathogen (Delgado‐Baquerizo et al., 2020). A field‐warming experiment showed that the abundance of the genus *Alternaria* increases with warming and its importance grows under climate‐change scenarios (Delgado‐Baquerizo et al., 2020).

*Alternaria* species adopt a wide variety of lifestyles. They mostly live as saprophytes in soil and decaying plant material (e.g., Thomma, 2003). There are *Alternaria* species, especially *A. alternata*, that cause allergies in humans or are pathogenic in immunocompromised patients. On plants, they are necrotrophic pathogens and cause economically relevant crop diseases and post‐harvest rots (e.g., Thomma, 2003). However, *Alternaria* can also live as an endophyte inside plants without causing disease (DeMers, 2022).

In this review, we focus on several *Alternaria* species that cause devastating diseases in the two solanaceous crops potato and tomato. We emphasize the diversity of *Alternaria* pathogens and diseases on these two hosts, which becomes especially evident through various taxonomic rearrangements and the ongoing progress in the fields of genetics and genomics. Such an overview over the diversity of causal agents can help guide studies on the molecular underpinnings of the plant–pathogen interaction, which we discuss in the second part of this review.

## 2 *ALTERNARIA* DISEASES ON POTATO AND TOMATO

**FIGURE 1:**
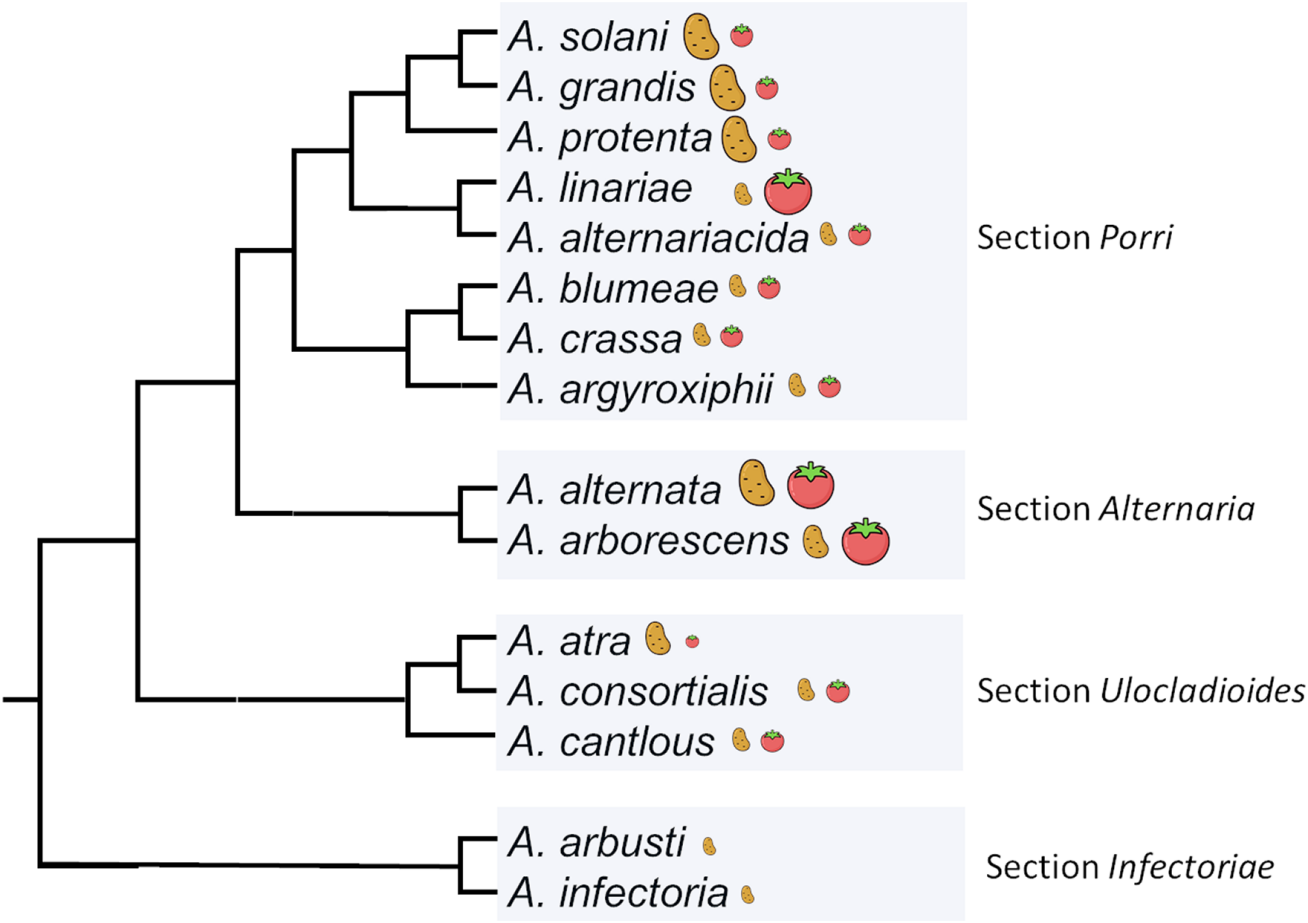
Phylogenetic tree of *Alternaria* spp. infecting tomato and potato. The relationships of the sections are based on Woudenberg et al. (2013) and Li et al. (2022). The relationships within section *Porri* are based on Woudenberg et al. (2014) and within section *Ulocladioides* on Woudenberg et al. (2013). The size of the tomato and potato symbols roughly represents the relative importance of the pathogens on both hosts according to the literature reviewed in this manuscript. Most species are reported to cause foliar diseases that can be referred to as early blight or leaf sporulation. *A. arborescens* is generally assumed to cause tomato stem canker. Tomato and potato icons by Rikas Dzihab.

Historically, researchers described early blight (EB) caused by *A. solani* and brown spot (BS) caused by *A. alternata*. Both pathogens occur together on the same plant and the initial stages of the symptoms are difficult to differentiate. Therefore, Vandecasteele et al. (2018) call the two diseases a disease complex, abbreviated as EB/BS. However, many more *Alternaria* species can be involved in this disease complex (Vandecasteele et al., 2018) and the taxonomic rearrangements in the genus *Alternaria* confuse species identification (see also methods for identification, Box 1). Therefore, we abbreviate it as EBDC for early blight disease complex. In the following section, we describe the different *Alternaria* pathogens infecting tomato and potato (Figure 1). Although all stages of plant growth and all plant organs including tomato fruit and potato tubers can be infected, we will focus on foliar symptoms without discussing post‐harvest diseases.

BOX 1 **Identification of *Alternaria* species**

The genus *Alternaria* underwent plenty and frequent taxonomic revisions (e.g., Lawrence et al., 2015; Li et al., 2022; Woudenberg et al., 2013), which can hinder or confuse species identification, for example, when public sequence databases are not updated (Dettman & Eggertson, 2021). Taxonomic revisions are still an ongoing process, not all sections are resolved (Dettman et al., 2023; Li et al., 2022) and novel species and sections are likely to be described when new habitats are investigated (Li et al., 2022).

Before molecular technologies were readily available, researchers classified *Alternaria* using morphological traits like colony and conidia characteristics (Lawrence et al., 2015). However, the widely used conidia shape is variable and depends on growth conditions, which leads to incorrect classifications (Thomma, 2003). Furthermore, these traits overlap between species and do not reflect the evolutionary relations (Dettman & Eggertson, 2021). In species formerly considered *Ulocladium*, morphological traits are also not reliable for species identification (Wang et al., 2010).

Several studies have investigated different molecular methods for distinguishing *Alternaria* species, for instance, random amplified microsatellites (RAMS) (Guo et al., 2004), restriction fragment length polymorphism (RFLP) (Adachi et al., 1993; Tanabe et al., 1989), random amplified polymorphic DNA (RAPD) (Morris et al., 2000; Roberts et al., 2000), amplified fragment length polymorphism (AFLP) (Somma et al., 2011), selective subtractive hybridization (Roberts et al., 2012), sequence‐characterized amplified genomic regions (SCAR) (Andrew et al., 2009; Stewart, Andrew, et al., 2013). As RAPD characterizes random priming sites across the entire genome, it can provide better resolution compared to the sequencing of some functional genes (Pinto & Patriarca, 2017).

The rRNA internal transcribed spacer (ITS) region is commonly sequenced as a barcode marker in fungal studies (Schoch et al., 2012), but in *Alternaria*, ITS and even additional commonly employed housekeeping genes do not have the power to discriminate between species (e.g., Dettman & Eggertson, 2021; Li et al., 2022; Woudenberg et al., 2013; Woudenberg et al., 2015).

According to Hong et al. (2005), the major allergen Alt a 1 facilitates identification to the species level, but later studies showed that it is not sufficient within some sections like the small‐spored section *Alternaria* (Dettman & Eggertson, 2021; Hong et al., 2005; Woudenberg et al., 2015). When using molecular barcode markers, a combination of several loci is necessary. The most commonly used markers are ITS, *GAPDH*, *RPB2*, *TEF1* and *Alt a 1* (e.g., Kokaeva et al., 2022; Woudenberg et al., 2014), and most studies with a multilocus phylogeny employ these five or subset of them, with or without additional, less common loci like *endoPG*, *histone H3*, *calmodulin* and *OPA 10–2* (e.g., Adhikari et al., 2020; Bessadat et al., 2021; Ding et al., 2019; Landschoot, Vandecasteele, Carrette, et al., 2017; Woudenberg et al., 2015). Recently, Dettman et al. developed (Dettman & Eggertson, 2021, 2022) and tested (Dettman et al., 2023) new markers to achieve better identification of small‐spored *Alternaria*.

As modern sequencing technologies become more accessible, whole genome data gain importance in *Alternaria* taxonomy. Especially within the small‐spored section *Alternaria*, whole genomes are an important tool (Dettman & Eggertson, 2021; Woudenberg et al., 2015).

Nishikawa and Nakashima claim that morphological and molecular phylogenetic data should be complemented by experimental host ranges to achieve an integrated species recognition (Nishikawa & Nakashima, 2020). Genes for host‐specific toxins are subject to horizontal gene transfer and some *A. alternata* pathotypes spontaneously lose their pathogenicity as a consequence of losing their capability to produce the host‐specific toxin, so pathotype should not be employed as a character in the taxonomy of small‐spored *Alternaria* (Andrew et al., 2009; Pinto & Patriarca, 2017). Chemotaxonomy, using secondary metabolite profiling for species identification, showed promising results for some species groups like *A. infectoria* (Andersen et al., 2008; Andersen & Thrane, 1996; Kelman et al., 2020; Zwickel et al., 2018) but not for all sections of *Alternaria* (Andersen et al., 2015; Zwickel et al., 2018). Secondary metabolite profiles can be combined with morphological and molecular analyses for polyphasic taxonomy (Pinto & Patriarca, 2017). The study by Woudenberg et al. (2015) is also an example for the combination of methodological approaches, as it employed a multilocus phylogeny, whole genome data and transcriptomics (Woudenberg et al., 2015).

### 2.1 Early blight

**FIGURE 2:**
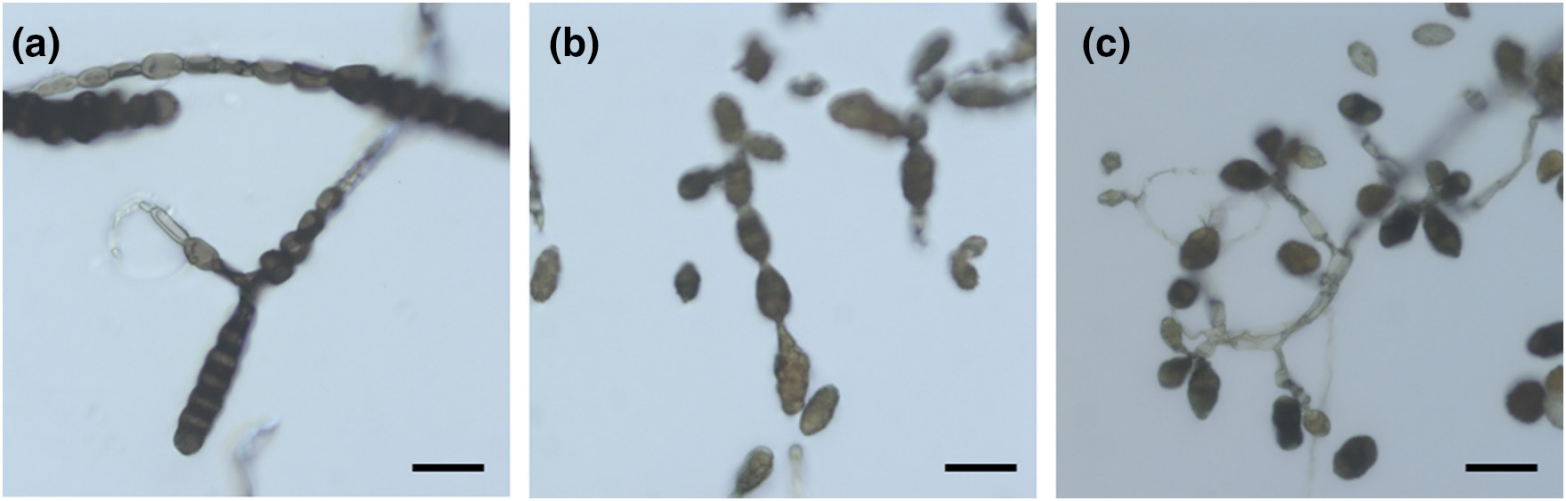
Conidia morphology. (a) *Alternaria* from section *Porri* are often referred to as large‐spored. (b) Small‐spored *Alternaria* from section *Alternaria* exhibit conidia growing in chains. (c) Species from section *Ulocladioides* have conidia that are comparable in size to other small‐spored species but grow in clusters rather than chains. Panels (b) and (c) are modified from Schmey et al. (2023). All three pathogens were collected from wild tomato species: (a) from *Solanum lycopersicoides*, (b) from *S. habrochaites* and (c) from *S. chilense*. Scale bars = 20 μm.

Early blight (EB) on potato and tomato is caused by large‐spored species of *Alternaria* (Figure 2). On potato, *A. solani* is considered the dominant pathogen (Tymon, Peever, et al., 2016). For a long time, all large‐spored *Alternaria* on plants of the *Solanaceae* family were determined as *A. solani* (Woudenberg et al., 2014). Simmons morphologically distinguished 22 large‐spored *Alternaria* species, including two species on potato, *A. solani* and *A. grandis*, and three other species on tomato, namely *A. tomatophila*, *A. cretica* and *A. subcylindrica* (Simmons, 2000). Woudenberg et al. (2014) disentangled the species in section *Porri* by molecular methods and described a third species, *A. protenta*, as an EB pathogen on potato (Woudenberg et al., 2014). Taxonomic rearrangements in the same paper synonymized five species names, including *tomatophila*, *cretica* and *subcylindrica*, under the name *A. linariae*. According to this study, tomato plants are also host to *A. protenta* and *A. alternariacida*. Recent work from Russia showed that *Alternaria alternariacida* can also infect potato plants (Kokaeva et al., 2022).

Potato and tomato are usually affected by different large‐spored *Alternaria* species (Woudenberg et al., 2014), for example, Rodrigues found all isolates from potato to be *A. grandis* and all isolates from tomato to be *A. tomatophila* (now *A. linariae*) (Rodrigues et al., 2010). However, all the above‐mentioned, large‐spored species appear capable of infecting both tomato and potato, as illustrated by reports from *A. grandis* on potato and tomato in Algeria (Bessadat et al., 2017) or *A. solani* and *A. linariae* on both host plants in Russia (Kokaeva et al., 2022).

Several other large‐spored *Alternaria* from the section *Porri* have also been reported on potato and tomato. *Alternaria blumeae* was reported on potato (Liu et al., 2019) and tomato (Htun et al., 2020). *Alternaria crassa* is reported on tomato and other hosts, but not potato (Bessadat et al., 2020; Peixoto et al., 2021), and an *A. crassa* isolate from another host was able to infect tomato (Peixoto et al., 2021). *Alternaria argyroxiphii* was reported on potato and even though it was not found on tomato plants, it was capable of infecting them under laboratory conditions (Zhao et al., 2023).

**FIGURE 3:**
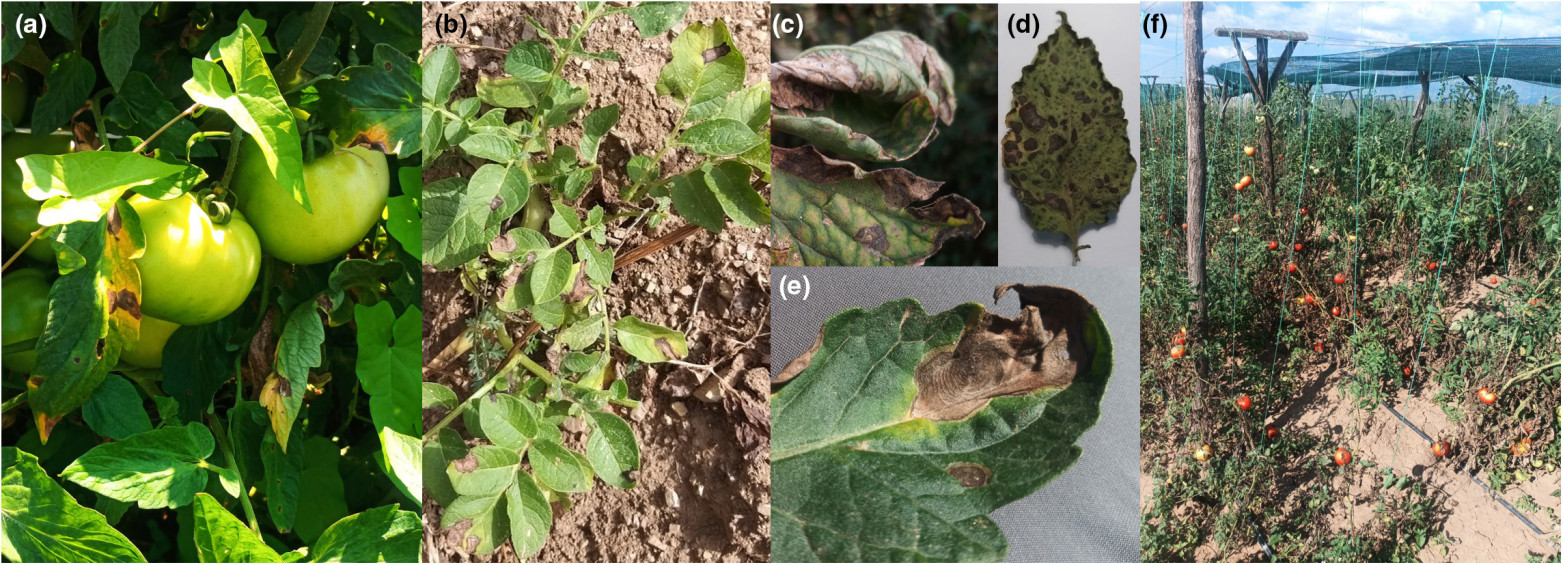
Symptoms. (a, b) Typical early blight symptoms on tomato and potato plants, respectively. (c) Severe early blight symptoms on an older tomato leaf. (d) Severe brown spot symptoms on a potato leaf. (e) Typical early blight lesions with clearly visible concentric rings. (f) Severe early blight disease complex infection on tomatoes, especially on the older, lower leaves. Pictures (b) and (d) by Gonne Clasen.

Early blight lesions start as small, brown spots and progress into dark brown to black lesions that usually develop concentric, target‐like rings (Agrios, 2005). They are relatively easy to identify, as they have a distinctive bull's‐eye‐shaped appearance with concentric rings (Figure 3) (Ding et al., 2019). Affected leaves become yellow and senescent until they dry up or fall off (Agrios, 2005). In severe cases, this can cause complete defoliation (Zhao et al., 2023).

### 2.2 Brown spot

Brown spot (BS) is caused by small‐spored *Alternaria* of section *Alternaria* (e.g., Wolters et al., 2019). Generally, *A. alternata* is reported as the causal agent for BS on potato and tomato (e.g., Ding et al., 2019), but some authors simply include *A. alternata* sensu lato as one of the species causing EB (e.g., Adhikari et al., 2017). Even though EB caused by large‐spored species is considered the dominant disease, small‐spored species are often recovered more frequently on potato (e.g., Tymon, Peever, et al., 2016). *A. alternata* f. sp. *lycopersici* is synonymous with *A. arborescens*. Both *A. alternata* and *A. arborescens* are often reported as causal agents of disease in tomato (e.g., El Gobashy et al., 2018). In susceptible tomato cultivars, *A. alternata* f. sp. *lycopersici* (now *A. arborescens*) causes stem canker disease, visible as dark brown cankers on the stems and necrosis of leaves (Witsenboer et al., 1992). Stem canker is distinct from EBDC due to important differences in pathogenesis, such as the prevalence of stem infections (Witsenboer et al., 1992), the use of host‐selective toxins (Meena et al., 2017), and the observation that jasmonic acid signalling increases susceptibility (Zhang et al., 2011), resulting in significant differences in the molecular mechanisms of infection. Landschoot, Vandecasteele, De Baets, et al. (2017) showed that some *A. arborescens* isolates can also infect potato crops.

Brown spot disease starts with small brown spots that are dispersed all over the leaf surface (Droby et al., 1984). Brown spot lesions are smaller than EB lesions and range from dots up to 10 mm in diameter (Ding et al., 2019). They also do not develop concentric rings (Bessadat et al., 2021). The spots can occur at every growth stage of the plant. With disease progression, the lesions coalesce into larger necrotic areas with brown margins, which can eventually cause dried, senescent leaves (Fairchild et al., 2013).

As *A. alternata* can lead a saprophytic lifestyle, the isolation of this fungus from a lesion does not necessarily confirm its pathogenicity (Tymon, Peever, et al., 2016). Even though small‐spored *Alternaria* have been reported as causal agents of brown spot disease on potato since 1984 (Droby et al., 1984), some authors still consider it controversial whether *A. alternata* is just an opportunistic secondary invader (e.g., Adolf et al., 2020). Many studies proved that *A. alternata* alone can infect tomato and potato leaves (e.g., Belosokhov et al., 2017) and Schmey et al. (2023) showed that small‐spored *Alternaria* are true pathogens on wild tomato species (Schmey et al., 2023).

### 2.3 *Ulocladium* leaf spot and other small‐spored *Alternaria*

The genus *Ulocladium* became a part of the genus *Alternaria* (Woudenberg et al., 2013). Several former *Ulocladium* species are known to cause symptoms on tomato and potato. *Alternaria atra*, formerly *Ulocladium atrum*, causes leaf spot or leaf blight on potato plants and is a serious disease of potato in Iran (Nasr‐Esfahani et al., 2021). The disease starts with dark brown or black lesions on the edges of the leaves that develop into irregular patches covering the whole leaf or even the whole plant (Esfahani, 2018). Interestingly, this pathogen has been investigated as a biocontrol agent, for example, against *Botrytis cinerea* (Elead et al., 1994).

*A. consortialis* belongs to the *Alternaria* section *Ulocladioides* and is the new name for *Ulocladium consortiale* and *Stemphylium consortiale* (Woudenberg et al., 2013). *A. consortialis* was reported to cause brown spots without concentric rings on potato leaves (Rich, 2013), while *Stemphylium consortiale* was reported pathogenic on tomato (Rich, 2013). Bessadat et al. (2017) reported *A. consortialis* to be pathogenic on tomato in Algeria. On potato tubers, however, it is not able to cause infection alone, only to make infections of *A. solani* more severe (Douglas, 1973).

*Ulocladium cantlous* became *Alternaria cantlous* (Woudenberg et al., 2013). Wang et al. (2010) originally described it from *Cucumis* sp. in China (Wang et al., 2010). On potato, it was first reported in 2016 (Amini et al., 2016) and on tomato in 2018 (Bessadat et al., 2018). Several other small‐spored species have been reported as leaf spot diseases on tomato and or potato. *A. arbusti* is a member of section *Infectoriae* (Lawrence et al., 2013, 2014; Woudenberg et al., 2013). It infects potato crops, especially when the potato leaves are wounded. However, it is a less aggressive pathogen than *A. solani* or *A. arborescens* because it is less capable of colonizing host tissue (Tymon, Cummings, et al., 2016). *A. infectoria* (also section *Infectoriae*) was found on potato crops in Russia and was proven to cause infection on potato (Belosokhov et al., 2017; Orina et al., 2010).

## 3 *ALTERNARIA* DIVERSITY ON POTATO AND TOMATO

Seeing the complexity of *Alternaria* diseases on both tomato and potato, many studies have tried to investigate the diversity of EBDC pathogens within and between fields on the species and subspecies levels. Initially, the studies used random amplified polymorphic DNA (RAPD), amplified fragment length polymorphism (AFLP) and microsatellites. Over the last 10 years, an increasing number of studies have sequenced gene loci, as sequencing technology has become more affordable. Nowadays, even whole genome sequencing is applied to investigate the diversity of *Alternaria* on potato (Einspanier et al., 2022).

As early as 1992, Petrunak and Christ (1992) used isozymes to study the diversity of *A. solani* and *A. alternata* on potato and other hosts in eight states of the United States. The resulting groups corresponded to pathogen species, but not geographic origin. Almost all studies since then have reported comparable results. A RAPD study by Weir et al. (1998) found differences between samples from the United States compared to non‐US samples, but also not on a smaller geographic scale within the United States (Weir et al., 1998). All kinds of diversity studies on *Alternaria* from both potato and tomato report that they did not find any geographic clusters/no correlation between genetic clusters and their geographical origin. All over the world, studies find that the investigated *Alternaria* genotypes are widespread (United States: Adhikari et al., 2021; Ding et al., 2019; Morris et al., 2000; Petrunak & Christ, 1992; Weir et al., 1998; South Africa: van der Waals et al., 2004; India: Prakash et al., 2022; Upadhyay et al., 2019; Varma et al., 2007; Brazil: Lourenço et al., 2011; China: Meng et al., 2015; Russia: Kokaeva et al., 2022). A study using whole genome sequencing on 48 isolates from five regions in four European countries defined seven major genotypes using principal component analysis (PCA) and genome‐structure‐based approaches. It reported that these genotypes are spread all over Europe; however, the sample set was too small to see whether there are tendencies of certain genotypes occurring with a preference in one of the regions (Einspanier et al., 2022).

Studies that also compared *Alternaria* pathogens between years found that genetic groups or haplotypes occur in several years. Weber and Halterman (2012) found seven RAPD profiles in two consecutive years (Weber & Halterman, 2012). Meng et al. (2015) detected 17.8% of multilocus genotypes in two and even 2% in three sampling years. Ding et al. (2019) used barcode sequencing to define genotypes and found the same genotypes five years apart. Adhikari et al. (2020) also sequenced barcodes and reported *A. linariae* or *A. solani* haplotypes in North Carolina and Wisconsin (United States) that had been found by Lourenço et al. (2009) in Brazil. Leiminger et al. (2013), however, reported pronounced genetic variability in the RAPD profiles of *A. solani* isolates from different years, indicating that *Alternaria* populations are indeed dynamic, but that it is important to find the right resolution.

The fact that identical genotypes can be retrieved from large geographic distances and over many years is consistent with the expectation of an asexual species (Meng et al., 2015). However, most studies report genotype diversities far higher than expected for purely asexual reproduction. This holds true for studies looking at *A. solani* (Einspanier et al., 2022; Leiminger et al., 2013; Lourenço et al., 2009, 2011; Upadhyay et al., 2019; van der Waals et al., 2004), *A. alternata* (Gherbawy et al., 2018; Meng et al., 2015) and studies that dealt with both large‐ and small‐spored species (Adhikari et al., 2020, 2021; Ozkilinc et al., 2018), as well as diverse *Alternaria* species from wild tomato hosts (Schmey et al., 2023). Possible evolutionary mechanisms leading to the high genetic diversity are mutation, gene flow and selection (Lourenço et al., 2011). We will discuss recombination and reproduction as mechanisms generating genetic diversity in a dedicated paragraph of this review.

Species within the large‐spored section *Porri* show lower genetic diversity at the common marker genes compared to species within the small‐spored section *Alternaria*. Ozkilinc et al. (2018) reported that isolates from section *Porri* had one or few genotypes per species while isolates of *A. alternata* and *A. arborescens* had many genotypes. A study on potato determined five *A. alternata* genotypes but only one *A. solani* genotype (Ding et al., 2019). According to Adhikari et al. (2020), *A. alternata* shows higher values for nucleotide diversity π and Watterson's θ compared to *A. linariae* or *A. solani*.

The relatively high genetic diversity is also illustrated by the fact that genetically different pathogen strains can be retrieved from the same lesion. This has been shown for *A. solani* (Kumar et al., 2008) and for *A. alternata* (Morris et al., 2000). Reasons for the higher diversity of *A. alternata* versus *A. solani* are not known but might be related to the broader host range of the former.

## 4 SEQUENCED GENOMES

*Alternaria* is a large fungal genus, there are many genome assemblies available, but only few of the sequenced pathogens were collected from potato and tomato host plants (Table 1). The National Center for Biotechnology Information (NCBI) database currently lists 169 *Alternaria* genome assemblies, but only one of these is from a potato host, two are from domesticated tomato hosts and one is from a wild tomato plant (https://www.ncbi.nlm.nih.gov/datasets/genome/?taxon=5598). The *Alternaria* genomes database (Dang et al., 2015) was an attempt to provide and visualize *Alternaria* genome annotation and comparison data but was not available anymore at the time of writing this review.

**TABLE 1:** Genome assembly statistics of selected *Alternaria* genomes.

Species	Isolate	Reference	Host plant	Genome size (Mb)	Number of scaffolds	G/C content (%)	Number of protein‐coding genes
*A. solani*	NL03003	Wolters et al. (2018)	Potato	32.78	10	51.32	NA
*A. solani*	HWC‐168	Zhang et al. (2018)	Potato	32.80	58 (NCBI), 61 (Zhang et al., 2018)	51.00	10,358 (Zhang et al., 2018), 11,951 (Wang, Xiao, et al., 2022)
*A. arborescens*	EGS 39–128	Hu et al. (2012)	Tomato	33.89	820	50.50	NA
*A. arborescens*	NRRL 20593	NCBI upload only	Tomato	33.59	701	51.00	NA
*A. alternata*	DZ	Lu et al. (2023)	Tobacco	34.11	11	50.95	11,556
*A. alternata*	Z7	Gai et al. (2021)	Citrus	34.36	12	50.99	12,067
*A. atra*	CS162	Bonthala et al. (2021)	Wild tomato	39.61	43	50.87	12,173

A gapless genome assembly is available for the *A. solani* isolate NL03003 from a potato field in the Netherlands (Wolters et al., 2018). It has a genome size of 32.8 Mb, which is approximately the same genome size reported in other studies. All 10 chromosomes have telomeres on both ends and the assembly represents the first finished genome of an *Alternaria* species (Wolters et al., 2018). The second *A. solani* genome on the NCBI database is more fragmented. This isolate HWC168 shows conserved long‐range synteny with the above‐mentioned *A. solani* NL03003 and was, for example, used to study candidate effector proteins (Wang, Xiao, et al., 2022). The other large‐spored species that cause early blight on tomato and/or potato have not been sequenced to date.

The NCBI database provides a plethora of genome assemblies for small‐spored *Alternaria* section *Alternaria*, but only two of these represent strains that were collected from a tomato plant, and none were collected from a potato host. The holotype of *A. arborescens* has been sequenced in a study investigating the conditionally dispensable chromosome (Hu et al., 2012). In 2020, another genome assembly for *A. arborescens* from tomato was uploaded to NCBI (https://www.ncbi.nlm.nih.gov/datasets/genome/GCA_013282825.1/). Both genomes have over 700 scaffolds. The *A. alternata* strain DZ was found on a tobacco host plant but provides a high‐quality reference genome for *A. alternata* (Lu et al., 2023). For the *A. alternata* isolate Z7, which causes brown spot of citrus, a high‐quality genome is available as well (Gai et al., 2021). A scaffold‐level genome assembly for *Alternaria atra* is available for an isolate that has been collected from a wild tomato plant (Bonthala et al., 2021). It has 43 scaffolds and a length of 39.6 Mb.

The gene density of *A. solani* (323 genes per Mb) is slightly lower than the gene density in *A. arborescens* (325 genes per Mb) (Zhang et al., 2018). According to Wang, Xiao, et al. (2022), the *A. solani* HWC‐168 genome has 11,951 predicted protein‐coding genes, of which 238 (2%) are candidate effector proteins. All of these candidate effectors have homologues in the *A. solani* NL03003 genome and are distributed over all 10 chromosomes. Other *Alternaria* species share up to 192 of these effector genes (Wang, Xiao, et al., 2022).

## 5 CONDITIONALLY DISPENSABLE CHROMOSOMES

Most *Alternaria* species have 10 essential chromosomes (Gai et al., 2021). These chromosomes may have experienced some rearrangements, but also show collinear and syntenic relationships between *Alternaria* genomes (Gai et al., 2021).

Some fungi, including several pathotypes of *A. alternata*, carry supernumerary chromosomes. They are also called accessory chromosomes or conditionally dispensable chromosomes (CDCs), because they are dispensable for growth, though they might be essential for pathogenicity (Johnson et al., 2001; Tsuge et al., 2016). Except for *A. alternata* tangerine pathotype strain Z7, which has two CDCs, the various pathotypes each contain one CDC (Wang et al., 2019). The size of the CDCs in these pathotypes ranges between 1.0 and 1.05 Mb in the tomato and strawberry pathotypes, respectively (Akagi et al., 2009; Hatta et al., 2002) to 1.9 Mb in the tangerine pathotype (Masunaka et al., 2005), but the Japanese pear pathotype carries a CDC of 4.1 Mb (Tanaka & Tsuge, 2000). The CDC sizes are comparable to the size of supernumerary chromosomes in other filamentous fungi, for example, 0.7 Mb in *Gibberella fujikuroi*, 1.2 or 2.0 Mb in different *Colletotrichum gloeosporioides*, or different supernumerary chromosomes of 1.5 Mb, 1.6 Mb but also 4.9 Mb in *Nectria haematococca* (Covert, 1998).

The biosynthetic genes for host‐specific toxins (HSTs, also called TOX genes) are located together as a single gene cluster in each of the *Alternaria* pathotypes (Tsuge et al., 2016). Gene clusters for secondary metabolites generally exist in a single set, but TOX clusters are often duplicated. TOX clusters, their location on CDCs and the intraspecies transfer of these CDCs have been reviewed in Tsuge et al. (2016).

The stem canker pathogen *A. arborescens* produces an HST called AAL. The biosynthetic gene cluster for AAL production is called ALT and located on a 1.0 Mb CDC (Kodama, 2019). There are probably two sets of ALT clusters (Tsuge et al., 2016). Interestingly, genetically different strains of this pathogen had identical sequences at two CDC genes and an identical CDC size, indicating that the essential chromosomes and the CDC probably have a different origin (Akagi et al., 2009). Hu et al. (2012) sequenced the *A. arborescens* genome and showed that the CDC was probably transferred through horizontal gene transfer by an unrelated fungus. They also found CDC genes under positive selection, which could indicate that they are candidate virulence factors (Hu et al., 2012). The presence of CDCs in the major large‐spored EBDC pathogens like *A. solani* has not yet been shown.

## 6 REPRODUCTION AND RECOMBINATION

Except for *A. infectoria*, sexual stages are not observed in *Alternaria* (e.g., DeMers, 2022). However, the diversity of *Alternaria* species and populations is far greater than expected for an asexual fungus. Several studies show possible recombination events when looking at genetic data in both large‐ and small‐spored *Alternaria* species. Two studies show indication for recombination in *A. alternata* from citrus (Stewart et al., 2014; Stewart, Thomas, et al., 2013) and Einspanier et al. (2022) found possible signatures of recombination when analysing the full genome of *A. solani* isolates. The observed diversity might result from cryptic sexuality, from a parasexual cycle, but might also have other causes.

One argument for cryptic sexual reproduction is the occurrence of both mating types. The two mating‐type regions are so diverged in fungi that they are called idiomorphs instead of alleles (Taylor et al., 2015). In *A. alternata*, each isolate has only one mating type, but both mating types are found in the species (Berbee et al., 2003). According to Stewart et al. (2011), both mating types are routinely recovered and Meng et al. (2015) showed that both mating types occur in equal frequencies in *A. alternata* in potato. Also, Armitage et al. (2020) found both mating types in a 1:1 ratio in both the *A. alternata* clade and the *A. arborescens* clade. Furthermore, *A. alternata* isolates with opposite mating types shared identical ITS sequences, meaning that if sexuality was lost, it was lost after the shared ITS substitutions (Berbee et al., 2003). As apple pathotype isolates can have either of the mating types, some (sexual or parasexual) genetic exchange must have taken place after the evolution of the CDC (Armitage et al., 2020). The *A. alternata* mating‐type genes are functional by heterologous expression (Arie et al., 2000) and also signs of purifying selection at the MAT1‐1 locus and biased codon usage can be interpreted as indications for sexual recombination in the recent past, cryptically in the present, or that MAT1‐1 has another cellular function (Stewart et al., 2011). In *A. solani* and *A. linariae*, the situation is similar: each isolate has one mating type and both mating types can be found in the species. However, Gannibal et al. (2014) observed a bias, as most *A. solani* isolates had mating type MAT‐1‐1 while most *A. linariae* isolates had MAT1‐2, which indicates the dominance of clonal reproduction.

Sometimes, repeat‐induced point mutations (RIP) are interpreted as signals of sexuality, because they would not be expected in asexual species. However, RIP‐like mutations have also been found in species that are thought to be asexual (Hane et al., 2015). van Wyk et al. (2021) classified *A. solani* as ‘Repeat‐Induced Point Class 4’, indicating moderate RIP levels and large RIP‐affected regions (LRARs) constituting a certain proportion of the genome.

The observed recombination could also stem from the parasexual cycle. Anastomoses are common in *A. solani* and heterokaryosis would be possible from a cytological standpoint (Stall, 1958). Anastomosis has also been described for *A. alternata* (Huang et al., 1996). The occurrence of vegetative compatibility, also referred to as mycelial compatibility, is a prerequisite for the parasexual cycle and therefore often interpreted as evidence or a hint for parasexual recombination. *A. solani* and *A. grandis* from potato both show vegetative compatibility (Alvarenga et al., 2016; van der Waals et al., 2004). Zhao, Fan, et al. (2021) claimed heterozygous diploids of *A. solani* are common in nature, showed hyphal and nuclear fusion in *A. solani*, and confirmed the haploidization process of parasexuality. Note that heterokaryosis in *A. alternata* has only been shown after mutagenesis with a carcinogen (Tsuge et al., 1987) or UV light (Hadi, 2021).

As mentioned, the CDCs of *Alternaria* pathotypes were probably acquired by horizontal chromosome transfer (HCT). An impressive and most relevant example of this HCT is the CDC in the tomato pathotype, *A. arborescens*, which had identical sequences even in samples that differed in their core chromosome sequences (Akagi et al., 2009). Hu et al. (2012) then provided evidence that the CDC was likely acquired from an unrelated fungus.

In addition to the transfer of whole chromosomes via HCT, it is also possible that only genes or gene clusters are acquired, which is called horizontal gene transfer (HGT). Several HGT events probably happened in *A. alternata* from citrus, where the acquired genes have important functions for sporulation (Wang et al., 2019).

Both HCT and HGT are very important for plant‐pathogenic fungi including *Alternaria*, as they are important mechanisms to broaden the host ranges of these pathogens (Mehrabi et al., 2011). The genes for HST of *A. alternata* are all found on CDCs, which are absent in nonpathogenic isolates, highlighting the importance of HCT for pathogenicity and host specificity of the different pathotypes (e.g., Mehrabi et al., 2011). The CDC from tomato‐infecting isolates, which is necessary for the production of AAL toxin and therefore for the infection of tomato plants, could be transferred to an isolate of the *A. alternata* strawberry pathotype, which consequently became capable of infecting both tomato and strawberry plants (Akagi et al., 2009). However, the described experiment was done using a protoplast fusion experiment and the exact mechanism of how CDCs or genes are transferred remains to be elucidated (Mehrabi et al., 2011).

## 7 LIFE CYCLE

**FIGURE 4:**
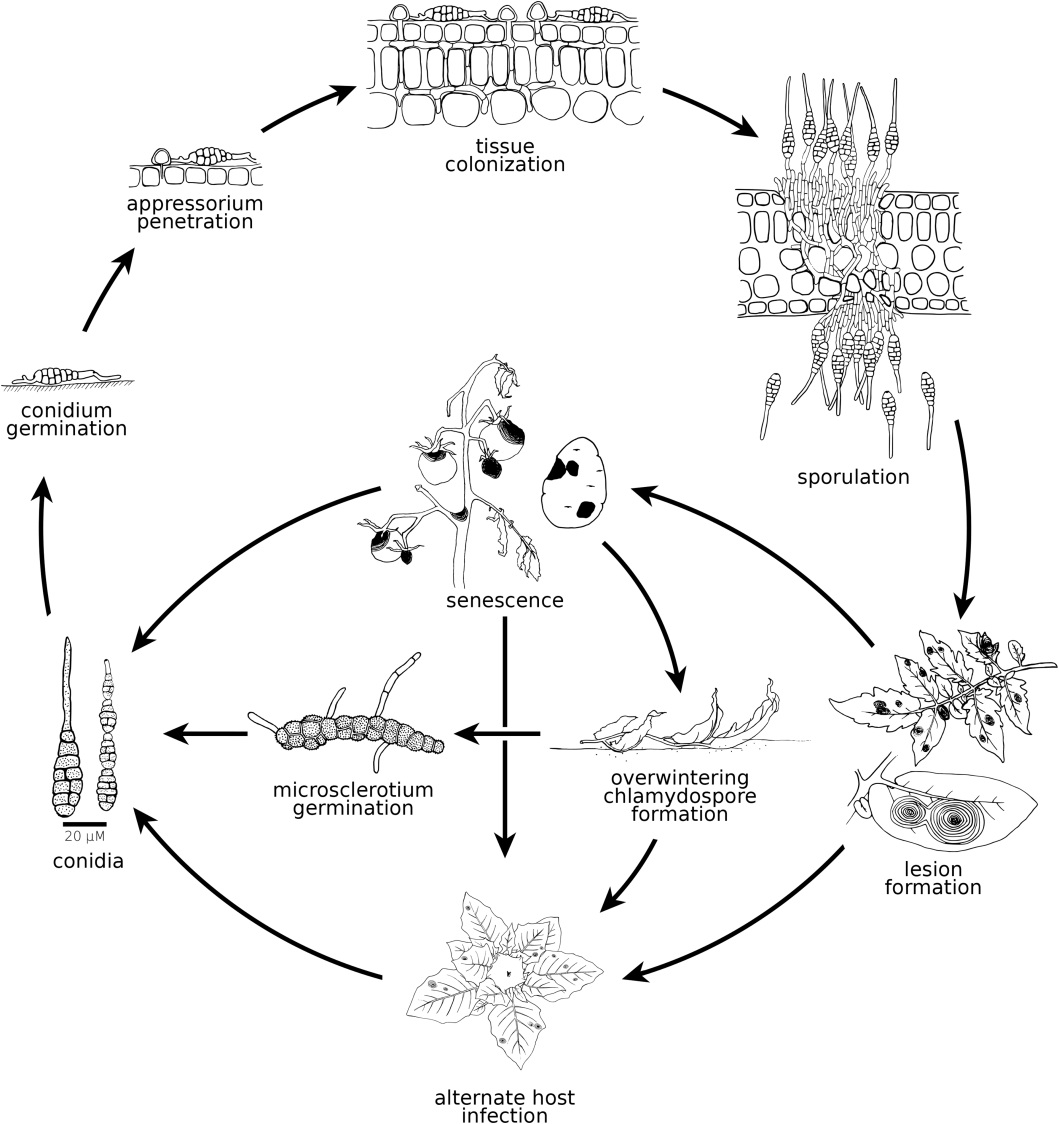
Life cycle representation of *Alternaria* spp. part of early blight disease complex (EBDC) in tomato and potato. Conidia, the primary reproductive propagules of EBDC, emerge from conidiophores (in *Alternaria* section *Porri* relatively large and non‐catenate, but smaller and catenate in *Alternaria* section *Alternata*). Conidia are carried by wind and rain to their hosts and germinate on the leaf surface. Host epidermis penetration occurs with or without appressoria. In favourable conditions, host tissue gets rapidly colonized by successive infestations. Sporulation at infection sites supports further host colonization and the spread of necrotic lesions that for some species adopt a bullseye appearance from the changes in growth rate as environmental conditions cycle between favourable and unfavourable over the course of days or weeks. The host can eventually defoliate and senesce as it is systematically colonized. For some species, infection also appears more prevalent in senescing tissue. In unfavourable conditions, hyphae in the necrotic lesions swell and thicken as they develop into chlamydospores. Chlamydospores can eventually coalesce into microsclerotia and overwinter to become soilborne inoculum for the next season. Conidiogenesis probably takes place directly on the microsclerotia, or from stem infections of a suitable host. The broad host range of EBDC allows successive waves in pathogenesis to spread to and from alternate hosts. Figure drawn by Andrea Goss.

No teleomorphs of EBDC‐causing *Alternaria* spp. are known (Meng et al., 2015). Thus, reproduction happens via multicellular and asexual conidia (Thomma, 2003). Conidia are released from their conidiophores by wind or rain, achieving high abundance in the air and soil (Figure 4; Agrios, 2005; Rotem, 1994). Optimal conditions for germination of EBDC conidia are 25°C, moistened host tissue, and 100% humidity (Thomidis et al., 2023). Germination usually occurs within 3 h, followed by a latent period preceding epidermal penetration that shortens with increasing virulence (Rotem, 1994). Entrance to host tissues is implemented by either invading wounds, thrusting penetration hyphae between epidermal cell interfaces via an appressorium, or by directly penetrating the epidermis using cell wall‐degrading enzymes (CWDEs) (Dita et al., 2007). Successful colonization leads to necrotic lesions after roughly 1–2 weeks, often circumscribed with a yellow halo of senescent tissue from the diffusion of fungal‐derived phytotoxins (Jones & Perez, 2023). Lesions produce additional conidia that systemically colonize the host to form secondary infections on leaves, stems, fruit, and tubers. Infections appear more prevalent in older, senescing tissues (Agrios, 2005). Primary lesions are often inconspicuous, and secondary sporulation leads to heavy infection later in the season (Zachmann, 1982). Due to the broad host range of EBDC, especially *A. alternata*, inoculum can originate from or spread to secondary hosts (DeMers, 2022). Conidiospores have thick, often melanized, cell walls and can probably survive in the soil for a certain amount of time (Chaerani & Voorrips, 2006; Lagopodi & Thanassoulopoulos, 1995). In the absence of suitable hosts, EBDC may enter a saprobic lifestyle (DeMers, 2022). After prolonged periods of unfavourable conditions late in the season, intercalary hyphae form chlamydospores that aggregate into microsclerotia (Basu, 1971; Lagopodi & Thanassoulopoulos, 1995). Microsclerotia tolerate adverse environmental conditions and overwinter in the soil until conditions become favourable for pathogenesis, exhibiting greater virulence in soil compared to any other cell type (Patterson, 1991).

## 8 PATHOGENESIS

**FIGURE 5:**
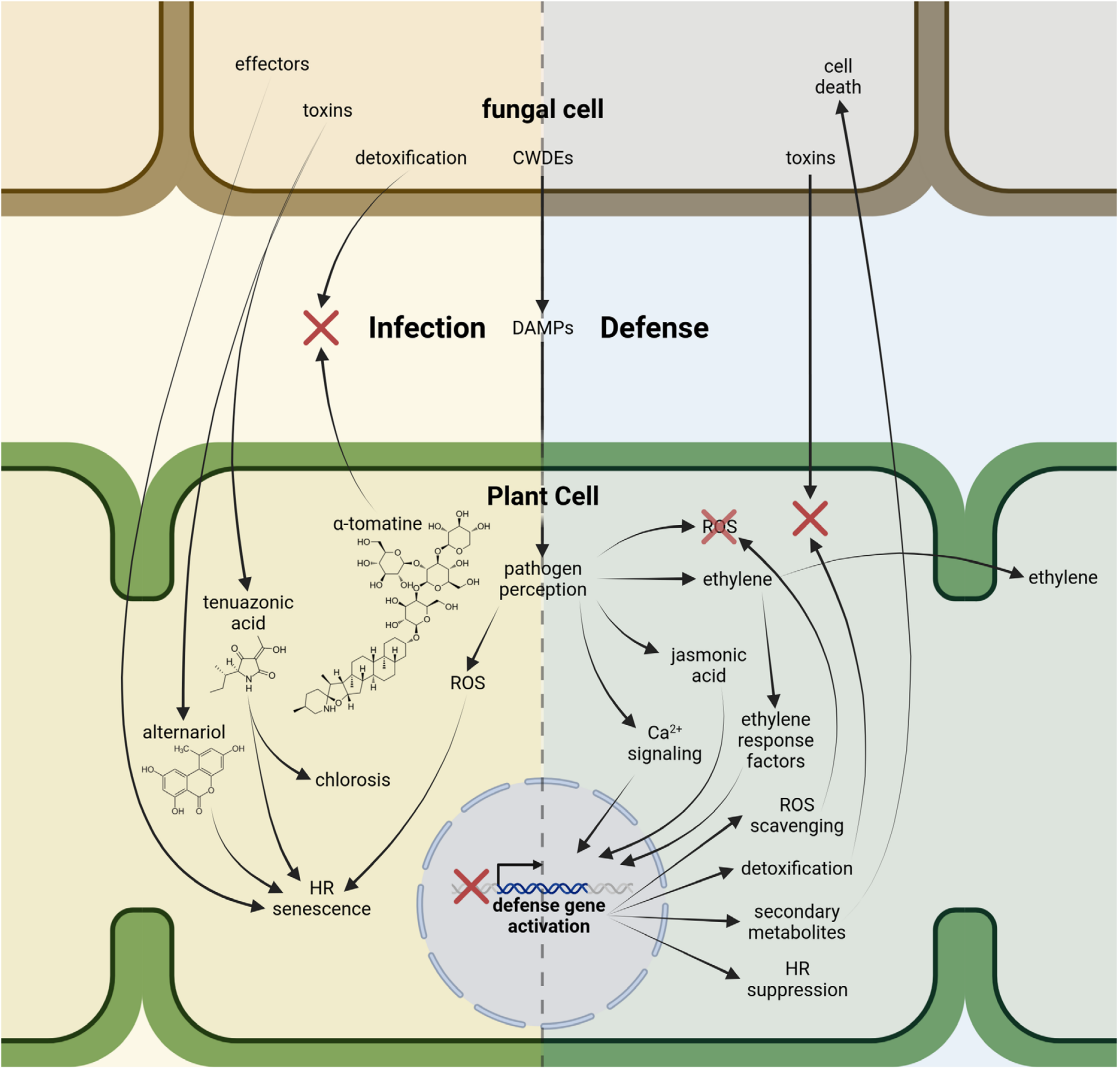
Infection and defence mechanisms. To initiate infection, the pathogens secrete cell wall‐degrading enzymes (CWDEs) that alert the host plant to pathogen presence, trigger a strong immune response that can lead to a hypersensitive response (HR), and remove physical barriers to host nutrients. Host phytoanticipins like α‐tomatine are detoxified. Pathogenic toxins are taken up by the host that induce chlorosis by inhibiting photosynthesis and promote HR by inducing a runaway oxidative burst of reactive oxygen species (ROS). Proteinaceous effectors might induce a HR by an undescribed method. To defend against early blight disease complex, calcium signalling and jasmonic acid biosynthesis indirectly contribute to the activation of plant defence gene transcription. Ethylene production activates ethylene response factors that directly activate defence gene transcription and diffuse to neighbouring cells to induce systemic defence responses. Defence genes contribute to defence by scavenging ROS to prevent a runaway oxidative burst, detoxification proteins to prevent cell death and chlorosis, production of secondary metabolites with direct antifungal activity to cause cell death pathogen tissues, and genes that further prevent HR directly or indirectly. The central dashed line separates the infection mechanisms (yellow background) from the defence mechanisms (blue background). Figure created with Biorender.

In typical necrotrophic pathogenesis, CWDEs remove physical barriers to host‐derived nutrients and trigger the immune response of the host sufficiently to induce a hypersensitive response (HR) of programmed cell death (Mengiste, 2012). HR and senescence increase susceptibility to necrotrophic pathogens like *Alternaria*, as the majority of its nutrition is assimilated from dead tissue as opposed to biotrophic pathogens that require extended parasitic periods on living tissue (Glazebrook, 2005). Caution should be taken against generalization of the role of HR in EBDC pathogenesis; although cell death‐promoting toxins and effectors are virulence factors (Wenderoth et al., 2019), reports from other hosts of *A. alternata* have shown HR is not necessary and sufficient to promote infection (Meng et al., 2018). The first physical barrier is the cuticle (Figure 5), and accordingly cutinases are highly upregulated during infection in the *A. solani–*potato pathosystem (Jiang et al., 2023). Cuticle lipids are potent damage‐associated molecular patterns (DAMPs) in tomatoes (Schweizer et al., 1996) and can contribute to HR, which has been shown in the *Alternaria brassicicola–Arabidopsis thaliana* pathosystem (Mang et al., 2009). The cell wall is the next physical barrier, and the virulence of small‐spored *Alternaria* to tomato correlates directly with the expression of pectinases and cellulases (Ramezani et al., 2019). Once cell death occurs and physical barriers are overcome, host cytoplasmic nutrients are assimilated, and the infection spreads.

Once inside host tissue, plant pathogenic *Alternaria* spp. secrete HSTs and non‐host‐specific toxins (NHSTs) (Figure 5) (Meena et al., 2017). In general, HSTs are acutely phytotoxic to their hosts, and NHSTs are mildly phytotoxic to both hosts and non‐hosts alike (Dalinova et al., 2020). The acute phytotoxicity of HSTs allows selective pressure on other virulence factors to relax, resulting in HSTs becoming the primary virulence mechanism, and a necessary component to establish infection (Akimitsu et al., 2014). Conversely, NHSTs cumulatively contribute to virulence and are not mandatory to establish an infection (Meena et al., 2017). The only known HST against any solanaceous taxon is the AAL toxin, which is produced by tomato pathogens in the *Alternaria arborescens* species complex, formerly known as *A. alternata* f. sp. *lycopersici* (Brandwagt et al., 2000). HSTs for EBDC are not known.

*Alternaria* spp. produce over 70 described secondary metabolites (Arcella et al., 2016). Many of them are phytotoxic NHSTs and reviewed in detail (Dalinova et al., 2020). No exhaustive list of relevant NHSTs and their myriad of derivatives such as sulphate conjugates have been published (Soukup et al., 2016). The following provides a brief overview of the major types: the dibenzo‐α‐pyrones alternariol (AOH), alternariol‐9‐*O*‐methyl ether (AME), and altenuene are virulence factors in tomatoes and cause necrotic lesions via a largely unknown mode of action (MoA) (Wenderoth et al., 2019). This MoA probably involves a combinatorial effect of AOH‐induced mitochondrial apoptosis signalling (Bensassi et al., 2012) and AME‐induced photosynthesis inhibition (Demuner et al., 2013). The perylene quinone derivatives altertoxin I and II, alterperylenol, and stemphyltoxin III are among the most phytotoxic (Visconti et al., 1989), mutagenic (Stack & Prival, 1986), and cytotoxic (Del Favero et al., 2018) EBDC NHSTs, but they have the least detection frequency in the food chain due to rapid detoxification by whole tomato fruits and low thermal stability (Puntscher & Marko, 2019). The tetrahydroanthraquinones altersolanol A, altersolanol B, and dactylariol have broad‐spectrum phytotoxicity with necrotic lesions occurring at lower concentrations in potato than tomato (Holenstein & Stoessl, 1983; Suemitsu et al., 1984). The MoA is not precisely elucidated, but there is an indication that the diversion of mitochondrial electrons to generate reactive oxygen species (ROS) plays a significant role (Haraguchi et al., 1996). Solanopyrones, especially A and D, induce necrotic lesions in hosts and non‐hosts via an unknown MoA. However, they are not necessary for virulence, possibly due to functional redundancy with other NHSTs (Ichihara et al., 1983; Kim et al., 2015). The tetrameric acid tenuazonic acid is probably the most acutely phytotoxic NHST of small‐spored *Alternaria* in the EBDC (Meena et al., 2016; Visconti et al., 1989). This acid inhibits the photosystem II (Chen et al., 2008). This MoA gives it a very wide phylogenetic range among which solanaceous plants are among the most tolerant (Zhou et al., 2019). The cyclic peptide tentoxin is also produced by small‐spored *Alternaria* in the EBDC and inhibits photophosphorylation by binding to F1‐ATPase (Groth, 2002). However, it only induces necrosis in potato and not in tomato (Edwards et al., 1987). The phenolic alkene alternaric acid is produced by large‐spored *Alternaria* in the EBDC and has broad‐spectrum phytotoxicity via an unknown MoA with herbicide potential similar to glyphosate (Israel et al., 2022). Tomato and potato are particularly susceptible to alternaric acid, which causes necrotic lesions similar to infection lesions caused by large‐spored *Alternaria* (Brian et al., 1952). All above‐mentioned NHST MoAs involve inducing oxidative stress to produce ROS. The cumulative effect of ROS‐evolving MoAs induces runaway immune signalling and HR. Indeed, EBDC virulence is well‐correlated with the phytotoxicity of its particular NHST cocktail (Shahbazi et al., 2011).

## 9 PROTEINACEOUS EFFECTORS

Pathogenic effectors are small, secreted proteins produced by pathogenic microbes that manipulate host cell structures and functions to facilitate infection and trigger defence responses. Effectors can be recognized by specific plant resistance (R) proteins leading to a rapid defence response in the processes of effector‐triggered immunity (ETI). Phytotoxins have been widely considered as the effector of *Alternaria* spp., especially HSTs (Akimitsu et al., 2014; Meena et al., 2017; Tsuge et al., 2013). However, hundreds of candidate effector proteins (CEPs) have been predicted for the 29 *Alternaria* spp. whose genomes have been sequenced, with 192 CEPs of *A. solani* having orthologues in *A. alternata* (Wang, Xiao, et al., 2022), highlighting the similarity of pathogenesis between the two EBDC taxa. To date, four *A. solani* CEPs have experimentally validated. During the infection of potato, expression of CEPs increases, and CEP deletion mutants have impaired virulence. Transient expression of CEPs in tomato leads to the development of necrotic lesions and the expression of host senescence genes (Wang, Xiao, et al., 2022; Wang, Zhang, et al., 2022; Wang et al., 2023).

In a transcriptomics study of the *A. solani–*potato pathosystem, 137 CEPs with domains for pectate lyases, cutinase, xylanase, glycoside hydrolase, and Nep1‐like proteins were significantly upregulated during late‐stage infections, and predicted to be proteinaceous effectors with high homology to pathogenicity‐related proteins (Jiang et al., 2023). EBDC proteinaceous effectors are a fascinating field of research as the molecular mechanisms of these effectors are yet to be uncovered, and it is likely that the 137 potato‐related CEPs have several mechanisms.

## 10 HOST DEFENCE

The plant immune system has been reviewed in detail numerous times (Jones & Dangl, 2006; Mengiste, 2012; Wang, Pruitt, et al., 2022). In brief, plants use a two‐tiered detection system consisting of extracellular pattern‐recognition receptors (PRRs) and cytoplasmic nucleotide‐binding domain leucine‐rich‐repeat containing receptors (NLRs) to identify microbial threats and regulate the two defence response layers known as pattern‐ and effector‐triggered immunity (PTI, ETI). While biotrophic pathogens must evolve host defence suppression mechanisms, necrotrophic pathogens like EBDC probably intentionally overstimulate host defence responses to induce HR or senescence (Glazebrook, 2005). Plant immune systems mirror this bipolar spectrum of bio‐ and necrotrophic nutrition in the mutual antagonism of the two major defence hormones salicylic acid (SA) and jasmonic acid (JA) (Thaler et al., 2012). SA and JA regulate defence responses to bio‐ and necrotrophs, respectively (Robert‐Seilaniantz et al., 2011). Strict control of SA biosynthesis is essential for proper defence against EBDC; when both SA and JA levels are elevated, SA signalling takes precedence, and SA generally promotes HR as an effective strategy against biotrophs (Caarls et al., 2015). Ethylene (ET) plays a significant role in necrotroph defence as ET signalling abolishes SA/JA antagonism (Leon‐Reyes et al., 2010), yielding priority to JA signalling pathways. Hormone signalling induces vigorous transcriptional responses, and although the specific role of JA in EBDC pathogenesis seems to be somewhat controversial (Brouwer et al., 2021; Sajeevan et al., 2023), JA seems to be a major transcriptional regulator of EBDC defence responses (Zheng et al., 2022).

Secondary metabolite biosynthesis is a crucial defence response to necrotrophs (Mengiste, 2012). To chemically defend against microbial pathogens, most plants induce antimicrobial phytoalexins or constitutively produce specialized phytoanticipins (VanEtten et al., 1994), such as glucosinolates for *Brassicaceae* (Halkier & Gershenzon, 2006), benzooxazinoids for *Poaceae* (de Bruijn et al., 2018), and saponins for *Solanaceae*, most notably the steroidal glycoalkaloids (SGAs, such as α‐tomatine) (Zhao, Zhao, et al., 2021). Comparative metabolic profiling of EBDC‐resistant and ‐susceptible wild tomato accessions showed differences in defence‐related metabolites like flavonoids, terpenoids, lignin, and SGAs in the resistant cultivar (Shinde et al., 2017). SGAs are generally antifungal by disrupting membrane stability (Osbourn, 1996), and JRE4 is the JA‐inducible master regulator of SGA biosynthesis (Nakayasu et al., 2018). Accordingly, methyl jasmonate (MeJA) is sufficient to induce antifungal compound biosynthesis, as tomato leaf extracts of MeJA‐treated plants inhibit conidiospore germination of EBDC (Kępczyńska & Król, 2012). This effect is not due to the abundant SGAs α‐tomatine and its derivatives alone, as EBDC is particularly resistant to them (Sandrock & VanEtten, 1998). This resistance is probably due to the ability of EBDC to detoxify the α‐tomatine derivatives (Osbourn, 1996), which both small‐ and large‐spored *Alternaria* spp. are capable of (Hoyos et al., 2023), implying the existence of more SGAs and other compounds in MeJA‐treated leaf extracts. Wild solanaceous plants are a veritable wellspring of antifungal SGAs, with broad‐spectrum antifungal tetraose SGAs conferring immunity to *Solanum commersonii* from EBDC (Wolters et al., 2023). This EBDC resistance locus has been successfully backcrossed into potato cultivars and confers broad resistance, also to other pathogens (Wolters et al., 2021), but the effects of tetraose SGAs to the environment and human ingestion are currently unknown. Metabolomics studies in EBDC remain scarce, but we found the alkaloid trigonelline is associated with successful EBDC resistance and confirmed its antifungal activity in a conidiospore germination inhibition assay (Hoyos et al., 2023), highlighting the ability of metabolomics to identify pathogenesis mechanisms from the host and pathogen.

Transcriptomics studies in the *A. solani*/potato pathosystem have highlighted the induction of peroxidases, terpene synthases, and JA/ET biosynthesis genes during EBDC infection, but questions remain about how a resistant cultivar is significantly more resistant than a susceptible cultivar (Sajeevan et al., 2023). We found that tomatoes treated with avirulent EBDC‐induced expression of D clade ethylene response factors (ERFs) that were highly influential in both co‐expression and gene regulatory networks associated with JA/ET biosynthesis, secondary metabolite biosynthesis and their transporters, detoxification, and calcium signalling (Tominello‐Ramirez et al., 2023). Conversely, tomatoes treated with virulent EBDC induced far fewer genes and no clear defence, either SA‐ or JA‐mediated. Proteomics studies have shown that resistant cultivars of tomatoes have increased protein levels related to protein stress, ROS scavenging, photosynthesis, posttranslational modification, secondary metabolite biosynthesis, and their transporters (Sadeghi et al., 2022).

Figure 5 provides a rough overview of the mechanisms involved in defence against EBDC. However, the molecular mechanisms of EBDC pathogenesis are currently far from resolved, but high‐throughput phenotyping is emerging as a promising method to identify quantitatively resistant cultivars to assist in genotype selection for other omics studies, or for plant breeding directly (Chakraborty et al., 2022).

## 11 RESISTANT GERMPLASM AND RESISTANCE BREEDING

Single (monogenic) resistance factors have not (yet) been identified. Adhikari et al. described several highly or moderately resistant tomato cultivars, including a few cultivars with relatively high leaf resistance, that rely on relatively few dominant genes and show moderately high heritability (Adhikari et al., 2017). They also described resistant accessions from wild tomato species such as *S. habrochaites, S. pennellii* and *S. peruvianum*, some of which have been used in crosses that led to successful identification of resistance‐associated quantitative trait loci (QTLs). Resistance in the before‐mentioned species had previously been described by Chaerani and Voorrips (2006), indicating that wild cultivars might be a good source for resistant germplasm. Using simple droplet infection assays, Chaerani et al. (2007) identified additional resistant accessions in other wild tomato species, such as *S. arcanum* and *S. chilense*. Yet, it should be noted that all *A. alternata* isolates tested in a study by Schmey et al. (2023) were able to infect detached leaves of various accessions of four different wild tomato species to a certain extent. Adhikari et al. (2017) pointed out that resistances identified in wild tomato species so far have not been very successfully used in resistance breeding programmes, possibly due to the relatively low effects of the individual QTLs and the difficulty of crossing species.

The situation in potato is similar. A study with close to 1000 tested cultivars, wild accessions and clones from different crossings showed a range of resistance properties, but no true full resistance. Interestingly, a link of resistance status with maturation time was expected, but not confirmed (Boiteux et al., 1995). This is different from a snapshot from Denmark with 38 cultivars, which showed that whereas resistance was clearly variable between cultivars, all late‐maturing cultivars were more resistant (Abuley et al., 2018), again indicating that EB resistance is linked to physiological processes in the plant and probably highly polygenic.

Quick spot infection assays can also be used to differentiate between resistant and susceptible potato species and cultivars (Wolters et al., 2019). Introgression lines of susceptible potato with wild potato relatives revealed that different wild potato species probably maintain different resistance mechanisms, some of which appear dominant and reliant on only one or few genes (Wolters et al., 2021).

Thus, QTL analyses could provide useful additional markers for resistance loci in both potato and tomato. Indeed, several recent studies showed that QTL mapping can provide insights on additional loci with roles in EBDC resistance in both tomato (Adhikari et al., 2023) and potato (Odilbekov et al., 2020; Xue et al., 2022).

## 12 EBDC MANAGEMENT AND FUNGICIDE RESISTANCE

As could be seen above, resistance to EBDC is linked to many physiological factors of the plant. Disease pressure is therefore highly cultivar‐dependent and, especially in potato, often linked to the maturity time. Early maturing varieties tend to be more susceptible because they retain older, senescent foliage that can serve as an easier entry point for the pathogen (Abuley et al., 2018). However, to our knowledge fully resistant cultivars do not exist. Whereas holistic control strategies are being discussed (Jindo et al., 2021), the complexity of the host–pathogen interaction makes fungicide application still the most effective measure against *Alternaria* spp. in an integrated plant protection strategy.

However, over the last decades loss of sensitivity and ultimately fungicide resistances have been reported for all major fungicide classes against EBDC. Two major fungicide groups target fungal respiration. Quinone‐outside inhibitors (QoIs), including, for example, azoxystrobin and pyroblostrobin, inhibit the mitochondrial respiration by preventing the electron transport chain of complex III (Bartlett et al., 2002). Another group of respiration inhibitors are the succinate dehydrogenase inhibitors (SDHIs), for example, boscalid. These also interfere with the electron transport chain, but at a different target site, namely succinate dehydrogenase, which is part of complex II (Avenot & Michailides, 2010).

The QoI resistance is mainly attributed to one specific point mutation in *A. solani* (F129L) and *A. alternata* (G134A) (Grasso et al., 2006; Pasche & Gudmestad, 2008). Studies from the United States showed a rapidly increasing level of resistance against QoIs, specifically azoxystrobin (Pasche & Gudmestad, 2008). The decreasing sensitivity was first observed two years after the fungicide became commercially available (Pasche et al., 2004) and Gudmestad et al. (2013) found the associated point mutation in 99% of the samples in 2010 and 2011. Leiminger et al. (2014) reported a similar development in Germany. QoIs were first registered as an early blight‐specific fungicide in 2007 and the first resistant isolates were found in 2009. In a later study from Sweden, Edin (2012) found the F129L mutation in nearly all tested isolates.

In contrast to QoI resistance, the SDHI resistance is associated with several point mutations. These mutations are distributed between subunit B (H278R and H278Y), subunit C (H134R), and subunit D (H133R and D123E) (Mallik et al., 2013). The first isolates with a mutation leading to boscalid resistance were found in Idaho in 2009 and 2010, fewer than five years after the fungicide was registered in the United States (Wharton et al., 2012). By 2014 and 2016, the occurrence of double mutations was confirmed in the United States and Belgium, respectively (Landschoot, Carrette, Vandecasteele, et al., 2017; Mallik et al., 2013). Studies from Nottensteiner et al. (2019) and Bauske et al. (2018) revealed at least one of the *sdh* mutations in 43% of German isolates and almost all US isolates, respectively. Whole genome sequencing of 48 *A. solani* isolates from all over Europe revealed that SDHI resistance mutations arose in different genetic backgrounds, indicating that SDHI resistance evolution happened multiple times independently, thus highlighting the evolutionary potential of *A. solani* (Einspanier et al., 2022).

The third fungicide group used against *Alternaria* spp. are demethylation inhibitors (DMIs). The mechanism of DMI resistance is associated with changed expression levels and possible mutation of the target site Cyp51 (Zhang et al., 2020). Overall, resistance against this group, which includes for example difenoconazole, is less prevalent than resistance against the respiration inhibitors. However, DMI‐resistant *A. alternata* isolates have been found on many crops (Avenot et al., 2014; Sun et al., 2021).

## 13 CONCLUSION

Compared to other plant pathogens, *Alternaria* spp. on potato and tomato remain relatively understudied. Yet, from a biological point of view, *Alternaria* warrant more study. Their genetic diversity, adaptability, and infection mechanisms provide novel context to our paradigms of fungal reproduction and pathogenesis. Research is essential for the development of sustainable strategies to manage EBDC and ensure the resilience of solanaceous crop production in agricultural lands of evolving pathogen pressure, loss of fungicide efficacy and greater climactic ranges. Understanding the complexities of EBDC and its impact on solanaceous crops, specifically potatoes and tomatoes, is a significant benefit for our agricultural systems. The diversity of *Alternaria* pathogens affecting these crops, across multiple species capable of infecting both hosts, as well as the various factors contributing to their genetic diversity, is crucial to a modern scientific understanding of asexual reproduction in fungi. Taxonomic revisions have complicated the identification of these pathogens and the research concerning them. We also emphasize the importance of a comprehensive understanding of *Alternaria* taxonomy for researchers of EBDC. The discovery of new species and revision of those already described is an important aspect of the ongoing investigations into the evolving nature of *Alternaria* spp. as pathogens and core members of the soil microbiota.

Research on *Alternaria* genetic diversity and pathogenesis has uncovered intriguing facets of their biology. Cryptic sexual reproduction, parasexual cycles, horizontal gene transfer, the potential for recombination, and genetic exchange within *Alternaria* populations all present a fascinating challenge to the assumption of strict asexuality for these fungi. The presence of both mating types and shared haplotypes suggests that sexual reproduction may be more prevalent than previously assumed. Further studies are warranted to elucidate the reproductive system of *Alternaria* fungi. The genetic diversity within *Alternaria* spp. and their ability to adapt to different host plants necessitates an in‐depth understanding of their genetic composition. As sequencing becomes more affordable, studies about the genetic diversity should employ full genomes. The availability of genome sequences has shed light on the role of CDCs and HCT in shaping the virulence of these fungi. However, the virulence beyond HSTs on CDCs, especially on fungi that probably do not carry a CDC like *A. solani*, should be studied further. The complex interactions between pathogens and hosts are influenced by various secondary metabolites, including HSTs and NHSTs, which are core components of the pathogenesis of EBDC. Tomatoes and potatoes employ PTI and probably ETI to trigger the defence hormone signalling pathways of JA and ET. Understanding these plant defence mechanisms is crucial for developing effective strategies to mitigate EBDC in solanaceous crops. Studies using crop wild relatives of tomatoes and potatoes have provided agronomic traits to elite crop varieties for yield, taste, and defence. Future protection and utilization of these key germplasm sources, coupled with omics studies of the molecular events that confer plant immunity to EBDC, represent the clearest path to sustained cultivation of solanaceous crops.

## Data Availability

Data sharing is not applicable to this article as no new data were created or analysed.

## References

[mpp13435-bib-0001] Abuley, I.K. , Nielsen, B.J. & Labouriau, R. (2018) Resistance status of cultivated potatoes to early blight (*Alternaria solani*) in Denmark. Plant Pathology, 67, 315–326.

[mpp13435-bib-0002] Adachi, Y. , Watanabe, H. , Tanabe, K. , Doke, N. , Nishimura, S. & Tsuge, T. (1993) Nuclear ribosomal DNA as a probe for genetic variability in the Japanese pear pathotype of *Alternaria alternata* . Applied and Environmental Microbiology, 59, 3197–3205. 16349060 10.1128/aem.59.10.3197-3205.1993 PMC182437

[mpp13435-bib-0003] Adhikari, P. , Oh, Y. & Panthee, D.R. (2017) Current status of early blight resistance in tomato: an update. International Journal of Molecular Sciences, 18, 2019. 28934121 10.3390/ijms18102019 PMC5666701

[mpp13435-bib-0004] Adhikari, T.B. , Ingram, T. , Halterman, D. & Louws, F.J. (2020) Gene genealogies reveal high nucleotide diversity and admixture haplotypes within three *Alternaria* species associated with tomato and potato. Phytopathology, 110, 1449–1464. 32202481 10.1094/PHYTO-12-19-0487-R

[mpp13435-bib-0005] Adhikari, T.B. , Muzhinji, N. , Halterman, D. & Louws, F.J. (2021) Genetic diversity and population structure of *Alternaria* species from tomato and potato in North Carolina and Wisconsin. Scientific Reports, 11, 17024. 34426589 10.1038/s41598-021-95486-6 PMC8382843

[mpp13435-bib-0006] Adhikari, T.B. , Siddique, M.I. , Louws, F.J. , Sim, S.‐C. & Panthee, D.R. (2023) Molecular mapping of quantitative trait loci for resistance to early blight in tomatoes. Frontiers in Plant Science, 14, 1135884. 37324699 10.3389/fpls.2023.1135884 PMC10267708

[mpp13435-bib-0007] Adolf, B. , Andrade‐Piedra, J. , Bittara Molina, F. , Przetakiewicz, J. , Hausladen, H. , Kromann, P. et al. (2020) Fungal, oomycete, and plasmodiophorid diseases of potato. In: Campos, H. & Ortiz, O. (Eds.) The potato crop: its agricultural, nutritional and social contribution to humankind. Cham: Springer International Publishing, pp. 307–350.

[mpp13435-bib-0008] Agrios, G.N. (2005) Plant pathology, 5th edition. Amsterdam, UA: Elsevier Academic Press.

[mpp13435-bib-0009] Akagi, Y. , Akamatsu, H. , Otani, H. & Kodama, M. (2009) Horizontal chromosome transfer, a mechanism for the evolution and differentiation of a plant‐pathogenic fungus. Eukaryotic Cell, 8, 1732–1738. 19749175 10.1128/EC.00135-09 PMC2772402

[mpp13435-bib-0010] Akimitsu, K. , Tsuge, T. , Kodama, M. , Yamamoto, M. & Otani, H. (2014) *Alternaria* host‐selective toxins: determinant factors of plant disease. Journal of General Plant Pathology, 80, 109–122.

[mpp13435-bib-0011] Alvarenga, T.V.M. , Ribeiro, S.R.R.D.P. , de Souza, E.A. , Pereira, F.d.C. & Pinto, C.A.B.P. (2016) Characterization of *Alternaria* isolates and reaction of potato genotypes to early blight. Ciência Rural, 46, 1783–1789.

[mpp13435-bib-0012] Amini, J. , Sepehrnoosh, S. & Abdollahzadeh, J. (2016) First report of *Alternaria cantlous* causing leaf spot on potato in Iran. Plant Disease, 100, 653.

[mpp13435-bib-0013] Andersen, B. , Dongo, A. & Pryor, B.M. (2008) Secondary metabolite profiling of *Alternaria dauci, A. porri, A. solani*, and *A. tomatophila* . Mycological Research, 112, 241–250. 18262401 10.1016/j.mycres.2007.09.004

[mpp13435-bib-0014] Andersen, B. , Nielsen, K.F. , Fernández Pinto, V. & Patriarca, A. (2015) Characterization of *Alternaria* strains from Argentinean blueberry, tomato, walnut and wheat. International Journal of Food Microbiology, 196, 1–10. 25498470 10.1016/j.ijfoodmicro.2014.11.029

[mpp13435-bib-0015] Andersen, B. & Thrane, U. (1996) Secondary metabolites produced by *Alternaria infectoria* and their use as chemotaxonomic markers. Mycotoxin Research, 12, 54–60. 23604651 10.1007/BF03192262

[mpp13435-bib-0016] Andrew, M. , Peever, T.L. & Pryor, B.M. (2009) An expanded multilocus phylogeny does not resolve morphological species within the small‐spored *Alternaria* species complex. Mycologia, 101, 95–109. 19271672 10.3852/08-135

[mpp13435-bib-0017] Arcella, D. , Eskola, M. & Gómez Ruiz, J.A. (2016) Dietary exposure assessment to *Alternaria* toxins in the European population. EFSA Journal, 14, e04654. 10.2903/j.efsa.2016.4654 PMC13088870 42006026

[mpp13435-bib-0018] Arie, T. , Kaneko, I. , Yoshida, T. , Noguchi, M. , Nomura, Y. & Yamaguchi, I. (2000) Mating‐type genes from asexual phytopathogenic ascomycetes *Fusarium oxysporum* and *Alternaria alternata* . Molecular Plant‐Microbe Interactions, 13, 1330–1339. 11106025 10.1094/MPMI.2000.13.12.1330

[mpp13435-bib-0019] Armitage, A.D. , Cockerton, H.M. , Sreenivasaprasad, S. , Woodhall, J. , Lane, C.R. , Harrison, R.J. et al. (2020) Genomics evolutionary history and diagnostics of the *Alternaria alternata* species group including apple and Asian pear pathotypes. Frontiers in Microbiology, 10, 3124. 32038562 10.3389/fmicb.2019.03124 PMC6989435

[mpp13435-bib-0020] Avenot, H.F. , Biggelaar, H.v.d. , Morgan, D.P. , Moral, J. , Joosten, M. & Michailides, T.J. (2014) Sensitivities of baseline isolates and boscalid‐resistant mutants of *Alternaria alternata* from pistachio to fluopyram, penthiopyrad, and fluxapyroxad. Plant Disease, 98, 197–205. 30708745 10.1094/PDIS-04-13-0459-RE

[mpp13435-bib-0021] Avenot, H.F. & Michailides, T.J. (2010) Progress in understanding molecular mechanisms and evolution of resistance to succinate dehydrogenase inhibiting (SDHI) fungicides in phytopathogenic fungi. Crop Protection, 29, 643–651.

[mpp13435-bib-0022] Bartlett, D.W. , Clough, J.M. , Godwin, J.R. , Hall, A.A. , Hamer, M. & Parr‐Dobrzanski, B. (2002) The strobilurin fungicides. Pest Management Science, 58, 649–662. 12146165 10.1002/ps.520

[mpp13435-bib-0023] Basu, P.K. (1971) Existence of chlamydospores of *Alternaria porri f. sp. solani* as overwintering propagules in soil. Phytopathology, 61, 1347.

[mpp13435-bib-0024] Bauske, M.J. , Mallik, I. , Yellareddygari, S.K.R. & Gudmestad, N.C. (2018) Spatial and temporal distribution of mutations conferring QoI and SDHI resistance in *Alternaria solani* across the United States. Plant Disease, 102, 349–358. 30673534 10.1094/PDIS-06-17-0852-RE

[mpp13435-bib-0025] Belosokhov, A.F. , Belov, G.L. , Chudinova, E.M. , Kokaeva, L.Y. & Elansky, S.N. (2017) *Alternaria* spp. and *Colletotrichum coccodes* in potato leaves with early blight symptoms. In: Sixteenth Euroblight workshop Aarhus, Denmark. PAGV–Special Report no. 18, pp. 181–190. Wageningen: Stichting Wageningen Research. Available from: https://agro.au.dk/fileadmin/23._A._Belosokhov‐p181‐190.pdf [Accessed 30th January 2024].

[mpp13435-bib-0026] Bensassi, F. , Gallerne, C. , Sharaf El Dein, O. , Hajlaoui, M.R. , Bacha, H. & Lemaire, C. (2012) Cell death induced by the *Alternaria* mycotoxin alternariol. Toxicology in Vitro, 26, 915–923. 22542754 10.1016/j.tiv.2012.04.014

[mpp13435-bib-0027] Berbee, M.L. , Payne, B.P. , Zhang, G. , Roberts, R.G. & Turgeon, B.G. (2003) Shared ITS DNA substitutions in isolates of opposite mating type reveal a recombining history for three presumed asexual species in the filamentous ascomycete genus *Alternaria* . Mycological Research, 107, 169–182. 12747328 10.1017/s0953756203007263

[mpp13435-bib-0028] Bessadat, N. , Berruyer, R. , Hamon, B. , Bataille‐Simoneau, N. , Benichou, S. , Kihal, M. et al. (2017) *Alternaria* species associated with early blight epidemics on tomato and other Solanaceae crops in northwestern Algeria. European Journal of Plant Pathology, 148, 181–197.

[mpp13435-bib-0029] Bessadat, N. , Hamon, B. , Bataillé‐Simoneau, N. , Chateau, C. , Mabrouk, K. & Simoneau, P. (2020) Occurrence of leaf spot disease caused by *Alternaria crassa* (Sacc.) rands on jimson weed and potential additional host plants in Algeria. The Plant Pathology Journal, 36, 179–184. 32296297 10.5423/PPJ.NT.01.2020.0003 PMC7143520

[mpp13435-bib-0030] Bessadat, N. , Hamon, B. , Bataille‐Simoneau, N. , Mabrouk, K. & Simoneau, P. (2018) *Alternaria* foliar diseases of solanaceous crops in Algeria: a multi‐species threat? Acta Horticulturae, 1257, 63–72.

[mpp13435-bib-0031] Bessadat, N. , Hamon, B. , Bataillé‐Simoneau, N. , Mabrouk, K. & Simoneau, P. (2021) Characterization of new small‐spored *Alternaria* species isolated from Solanaceae in Algeria. Life, 11, 1291. 34947822 10.3390/life11121291 PMC8704928

[mpp13435-bib-0032] Boiteux, L.S. , Reifschneider, F.J.B. , Fonseca, M.E.N. & Buso, J.A. (1995) Search for sources of early blight (*Alternaria solani*) field resistance not associated with vegetative late maturity in tetraploid potato germplasm. Euphytica, 83, 63–70.

[mpp13435-bib-0033] Bonthala, B. , Small, C.S. , Lutz, M.A. , Graf, A. , Krebs, S. , Sepúlveda, G. et al. (2021) ONT‐based draft genome assembly and annotation of *Alternaria atra* . Molecular Plant–Microbe Interactions, 34, 870–873. 33779266 10.1094/MPMI-01-21-0016-A

[mpp13435-bib-0034] Brandwagt, B.F. , Mesbah, L.A. , Takken, F.L.W. , Laurent, P.L. , Kneppers, T.J.A. , Hille, J. et al. (2000) A longevity assurance gene homolog of tomato mediates resistance to *Alternaria alternata f. sp. lycopersici* toxins and fumonisin B1. Proceedings of the National Academy of Sciences of the United States of America, 97, 4961–4966. 10781105 10.1073/pnas.97.9.4961 PMC18340

[mpp13435-bib-0035] Brian, P.W. , Elson, G.W. , Hemming, H.G. & Wright, J.M. (1952) The phytotoxic properties of alternaric acid in relation to the etiology of plant diseases caused by *Alternaria solani* (Ell. & Mart.) Jones & Grout. Annals of Applied Biology, 39, 308–321.

[mpp13435-bib-0036] Brouwer, S.M. , Brus‐Szkalej, M. , Saripella, G.V. , Liang, D. , Liljeroth, E. & Grenville‐Briggs, L.J. (2021) Transcriptome analysis of potato infected with the necrotrophic pathogen *Alternaria solani* . Plants, 10, 2212. 34686023 10.3390/plants10102212 PMC8539873

[mpp13435-bib-0037] Caarls, L. , Pieterse, C.M.J. & Van Wees, S.C.M. (2015) How salicylic acid takes transcriptional control over jasmonic acid signaling. Frontiers in Plant Science, 6, 170. 25859250 10.3389/fpls.2015.00170 PMC4373269

[mpp13435-bib-0038] Chaerani, R. , Groenwold, R. , Stam, P. & Voorrips, R.E. (2007) Assessment of early blight (*Alternaria solani*) resistance in tomato using a droplet inoculation method. Journal of General Plant Pathology, 73, 96–103.

[mpp13435-bib-0039] Chaerani, R. & Voorrips, R.E. (2006) Tomato early blight (*Alternaria solani*): the pathogen, genetics, and breeding for resistance. Journal of General Plant Pathology, 72, 335–347.

[mpp13435-bib-0040] Chakraborty, K.K. , Mukherjee, R. , Chakroborty, C. & Bora, K. (2022) Automated recognition of optical image based potato leaf blight diseases using deep learning. Physiological and Molecular Plant Pathology, 117, 101781.

[mpp13435-bib-0041] Chen, S. , Yin, C. , Dai, X. , Qiang, S. & Xu, X. (2008) Action of tenuazonic acid, a natural phytotoxin, on photosystem II of spinach. Environmental and Experimental Botany, 62, 279–289.

[mpp13435-bib-0042] Covert, S.F. (1998) Supernumerary chromosomes in filamentous fungi. Current Genetics, 33, 311–319. 9618581 10.1007/s002940050342

[mpp13435-bib-0043] Dalinova, A.A. , Salimova, D.R. & Berestetskiy, A.O. (2020) Fungi of the genera *Alternaria* as producers of biological active compounds and mycoherbicides. Applied Biochemistry and Microbiology, 56, 256–272.

[mpp13435-bib-0044] Dang, H.X. , Pryor, B. , Peever, T. & Lawrence, C.B. (2015) The *Alternaria* genomes database: a comprehensive resource for a fungal genus comprised of saprophytes, plant pathogens, and allergenic species. BMC Genomics, 16, 239. 25887485 10.1186/s12864-015-1430-7 PMC4387663

[mpp13435-bib-0045] de Bruijn, W.J.C. , Gruppen, H. & Vincken, J.‐P. (2018) Structure and biosynthesis of benzoxazinoids: plant defence metabolites with potential as antimicrobial scaffolds. Phytochemistry, 155, 233–243. 30218957 10.1016/j.phytochem.2018.07.005

[mpp13435-bib-0046] Del Favero, G. , Zaharescu, R. & Marko, D. (2018) Functional impairment triggered by altertoxin II (ATXII) in intestinal cells in vitro: cross‐talk between cytotoxicity and mechanotransduction. Archives of Toxicology, 92, 3535–3547. 30276433 10.1007/s00204-018-2317-6 PMC6290659

[mpp13435-bib-0047] Delgado‐Baquerizo, M. , Guerra, C.A. , Cano‐Díaz, C. , Egidi, E. , Wang, J.‐T. , Eisenhauer, N. et al. (2020) The proportion of soil‐borne pathogens increases with warming at the global scale. Nature Climate Change, 10, 550–554.

[mpp13435-bib-0048] DeMers, M. (2022) *Alternaria alternata* as endophyte and pathogen. Microbiology, 168, 001153. 35348451 10.1099/mic.0.001153 PMC9558358

[mpp13435-bib-0049] Demuner, A.J. , Barbosa, L.C.A. , Miranda, A.C.M. , Geraldo, G.C. , Silva, C.M.d. , Giberti, S. et al. (2013) The fungal phytotoxin alternariol 9‐methyl ether and some of its synthetic analogues inhibit the photosynthetic electron transport chain. Journal of Natural Products, 76, 2234–2245. 24245962 10.1021/np4005882

[mpp13435-bib-0050] Dettman, J.R. & Eggertson, Q. (2021) Phylogenomic analyses of *Alternaria* section *Alternaria*: a high‐resolution, genome‐wide study of lineage sorting and gene tree discordance. Mycologia, 113, 1218–1232. 34637684 10.1080/00275514.2021.1950456

[mpp13435-bib-0051] Dettman, J.R. & Eggertson, Q. (2022) New molecular markers for distinguishing the main phylogenetic lineages within *Alternaria* section *Alternaria* . Canadian Journal of Plant Pathology, 44, 754–766.

[mpp13435-bib-0052] Dettman, J.R. , Eggertson, Q.A. & Kim, N.E. (2023) Species diversity and molecular characterization of *Alternaria* section *Alternaria* isolates collected mainly from cereal crops in Canada. Frontiers in Microbiology, 14, 1194911. 37303811 10.3389/fmicb.2023.1194911 PMC10249498

[mpp13435-bib-0053] Ding, S. , Meinholz, K. , Cleveland, K. , Jordan, S.A. & Gevens, A.J. (2019) Diversity and virulence of *Alternaria* spp. causing potato early blight and brown spot in Wisconsin. Phytopathology, 109, 436–445. 30256185 10.1094/PHYTO-06-18-0181-R

[mpp13435-bib-0054] Dita, M.A. , Brommonschenkel, S.H. , Matsuoka, K. & Mizubuti, E.S.G. (2007) Histopathological study of the *Alternaria solani* infection process in potato cultivars with different levels of early blight resistance. Journal of Phytopathology, 155, 462–469.

[mpp13435-bib-0055] Douglas, D.R. (1973) Effect of *Ulocladium consortiale*, a nonpathogen, on the severity of tuber blight caused by *Alternaria solani* . American Potato Journal, 50, 353–356.

[mpp13435-bib-0056] Droby, S. , Dinoor, A. , Prusky, D. & Barkai‐Golan, R. (1984) Pathogenicity of *Alternaria alternata* on potato in Israel. Phytopathology, 74, 537–542.

[mpp13435-bib-0057] Edin, E. (2012) Species specific primers for identification of *Alternaria solani*, in combination with analysis of the F129L substitution associates with loss of sensitivity toward strobilurins. Crop Protection, 38, 72–73.

[mpp13435-bib-0058] Edwards, J.V. , Lax, A.R. , Lillehoj, E.B. & Boudreaux, G.J. (1987) Structure–activity relationships of cyclic and acyclic analogues of the phytotoxic peptide tentoxin. Journal of Agricultural and Food Chemistry, 35, 451–456.

[mpp13435-bib-0059] Einspanier, S. , Susanto, T. , Metz, N. , Wolters, P.J. , Vleeshouwers, V.G.A.A. , Lankinen, Å. et al. (2022) Whole‐genome sequencing elucidates the species‐wide diversity and evolution of fungicide resistance in the early blight pathogen *Alternaria solani* . Evolutionary Applications, 15, 1605–1620. 36330303 10.1111/eva.13350 PMC9624079

[mpp13435-bib-0060] El Gobashy, S.F. , Mikhail, W.Z.A. , Ismail, A.M. , Zekry, A. , Moretti, A. , Susca, A. et al. (2018) Phylogenetic, toxigenic and virulence profiles of *Alternaria* species causing leaf blight of tomato in Egypt. Mycological Progress, 17, 1269–1282.

[mpp13435-bib-0061] Elead, Y. , Köhl, J. & Fokkema, N.J. (1994) Control of infection and sporulation of *Botrytis cinerea* on bean and tomato by saprophytic bacteria and fungi. European Journal of Plant Pathology, 100, 315–336.

[mpp13435-bib-0062] Esfahani, M.N. (2018) Identification of *Ulocladium atrum* causing potato leaf blight in Iran. Phytopathologia Mediterranea, 57, 112–114.

[mpp13435-bib-0063] Fairchild, K.L. , Miles, T.D. & Wharton, P.S. (2013) Assessing fungicide resistance in populations of *Alternaria* in Idaho potato fields. Crop Protection, 49, 31–39.

[mpp13435-bib-0064] Gai, Y. , Ma, H. , Chen, Y. , Li, L. , Cao, Y. , Wang, M. et al. (2021) Chromosome‐scale genome sequence of *Alternaria alternata* causing *Alternaria* brown spot of citrus. Molecular Plant–Microbe Interactions, 34, 726–732. 33689393 10.1094/MPMI-10-20-0278-SC

[mpp13435-bib-0065] Gannibal, P.B. , Orina, A.S. , Mironenko, N.V. & Levitin, M.M. (2014) Differentiation of the closely related species, *Alternaria solani* and *A. tomatophila*, by molecular and morphological features and aggressiveness. European Journal of Plant Pathology, 139, 609–623.

[mpp13435-bib-0066] Gherbawy, Y. , Hussein, M.A. , Runge, F. & Spring, O. (2018) Molecular characterization of *Alternaria alternata* population isolated from upper Egyptian tomato fruits. Journal of Phytopathology, 166, 709–721.

[mpp13435-bib-0067] Glazebrook, J. (2005) Contrasting mechanisms of defense against biotrophic and necrotrophic pathogens. Annual Review of Phytopathology, 43, 205–227. 10.1146/annurev.phyto.43.040204.135923 16078883

[mpp13435-bib-0068] Grasso, V. , Palermo, S. , Sierotzki, H. , Garibaldi, A. & Gisi, U. (2006) Cytochrome *b* gene structure and consequences for resistance to Qo inhibitor fungicides in plant pathogens. Pest Management Science, 62, 465–472. 16688790 10.1002/ps.1236

[mpp13435-bib-0069] Groth, G. (2002) Structure of spinach chloroplast F1‐ATPase complexed with the phytopathogenic inhibitor tentoxin. Proceedings of the National Academy of Sciences of the United States of America, 99, 3464–3468. 11904410 10.1073/pnas.052546099 PMC122546

[mpp13435-bib-0070] Gudmestad, N.C. , Arabiat, S. , Miller, J.S. & Pasche, J.S. (2013) Prevalence and impact of SDHI fungicide resistance in *Alternaria solani* . Plant Disease, 97, 952–960. 30722567 10.1094/PDIS-12-12-1176-RE

[mpp13435-bib-0071] Guo, L. , Xu, L. , Zheng, W.‐H. & Hyde, K.D. (2004) Genetic variation of *Alternaria alternata*, an endophytic fungus isolated from *Pinus tabulaeformis* as determined by random amplified microsatellites (RAMS). Fungal Diversity, 16, 53–65.

[mpp13435-bib-0072] Hadi, H.W. (2021) Isolation of heterozygous diploid strain in *Alternaria alternata* fungus. Algerian Journal of Research and Technology, 5, 41–50.

[mpp13435-bib-0073] Halkier, B.A. & Gershenzon, J. (2006) Biology and biochemistry of glucosinolates. Annual Review of Plant Biology, 57, 303–333. 10.1146/annurev.arplant.57.032905.105228 16669764

[mpp13435-bib-0074] Hane, J.K. , Williams, A.H. , Taranto, A.P. , Solomon, P.S. & Oliver, R.P. (2015) Repeat‐induced point mutation: a fungal‐specific, endogenous mutagenesis process. In: van den Berg, M.A. & Maruthachalam, K. (Eds.) Genetic transformation systems in fungi, volume 2 fungal biology. Cham: Springer International Publishing, pp. 55–68.

[mpp13435-bib-0075] Haraguchi, H. , Abo, T. , Fukuda, A. , Okamura, N. & Yagi, A. (1996) Mode of phytotoxic action of altersolanols. Phytochemistry, 43, 989–992.

[mpp13435-bib-0076] Hatta, R. , Ito, K. , Hosaki, Y. , Tanaka, T. , Tanaka, A. , Yamamoto, M. et al. (2002) A conditionally dispensable chromosome controls host‐specific pathogenicity in the fungal plant pathogen *Alternaria alternata* . Genetics, 161, 59–70. 12019223 10.1093/genetics/161.1.59 PMC1462115

[mpp13435-bib-0077] Holenstein, J.E. & Stoessl, A. (1983) Metabolites of *Alternaria solani*, part IX: phytotoxicity of altersolanol A. Journal of Phytopathology, 108, 143–147.

[mpp13435-bib-0078] Hong, S.G. , Cramer, R.A. , Lawrence, C.B. & Pryor, B.M. (2005) Alt a 1 allergen homologs from *Alternaria* and related taxa: analysis of phylogenetic content and secondary structure. Fungal Genetics and Biology, 42, 119–129. 15670710 10.1016/j.fgb.2004.10.009

[mpp13435-bib-0079] Hoyos, L.M. , Wan, P.A. , Meng, C. , Kleigrewe, K. , Dawid, C. , Hückelhoven, R. et al. (2023) Untargeted metabolomics reveals PTI‐associated metabolites in tomato. *bioRxiv* [Preprint] doi: 10.1101/2023.06.15.544816 38164085

[mpp13435-bib-0080] Htun, A.A. , Aung, S.L.L. , He, L. , Liu, H.F. & Deng, J.X. (2020) First report of *Alternaria blumeae* causing leaf blight on tomato in Myanmar. Plant Disease, 104, 1871.

[mpp13435-bib-0081] Hu, J. , Chen, C. , Peever, T. , Dang, H. , Lawrence, C. & Mitchell, T. (2012) Genomic characterization of the conditionally dispensable chromosome in *Alternaria arborescens* provides evidence for horizontal gene transfer. BMC Genomics, 13, 171. 22559316 10.1186/1471-2164-13-171 PMC3443068

[mpp13435-bib-0082] Huang, S.‐L. , Itoh, Y. , Kohmoto, K. , Otani, H. & Kodama, M. (1996) Hyphal anastomosis and complementary growth of fused cells in *Alternaria alternata* . Mycoscience, 37, 1–13.

[mpp13435-bib-0083] Ichihara, A. , Tazaki, H. & Sakamura, S. (1983) Solanapyrones A, B and C, phytotoxic metabolites from the fungus *Alternaria solani* . Tetrahedron Letters, 24, 5373–5376.

[mpp13435-bib-0084] Israel, E.M. , Comas‐Barceló, J. , Slawin, A.M.Z. & Watson, A.J.B. (2022) Total synthesis and structure–activity relationship of alternaric acid delivers an herbicide vector. Nature Synthesis, 1, 987–995.

[mpp13435-bib-0085] Jiang, J. , Guo, X. , Tan, H. , Ding, M. , Liu, F. , Yang, Z. et al. (2023) Transcriptome sequencing leads to an improved understanding of the infection mechanism of *Alternaria solani* in potato. BMC Plant Biology, 23, 120. 36859112 10.1186/s12870-023-04103-3 PMC9976505

[mpp13435-bib-0086] Jindo, K. , Evenhuis, A. , Kempenaar, C. , Pombo Sudré, C. , Zhan, X. , Goitom Teklu, M. et al. (2021) Review: holistic pest management against early blight disease towards sustainable agriculture. Pest Management Science, 77, 3871–3880. 33538396 10.1002/ps.6320 PMC8451811

[mpp13435-bib-0087] Johnson, L.J. , Johnson, R.D. , Akamatsu, H. , Salamiah, A. , Otani, H. , Kohmoto, K. et al. (2001) Spontaneous loss of a conditionally dispensable chromosome from the *Alternaria alternata* apple pathotype leads to loss of toxin production and pathogenicity. Current Genetics, 40, 65–72. 11570518 10.1007/s002940100233

[mpp13435-bib-0088] Jones, J.D.G. & Dangl, J.L. (2006) The plant immune system. Nature, 444, 323–329. 17108957 10.1038/nature05286

[mpp13435-bib-0089] Jones, R.W. & Perez, F. (2023) Differential plant response to toxins and elicitor proteins released by the potato and tomato pathogens *Alternaria solani* and *Alternaria alternata* . Journal of Plant Pathology, 105, 21–28.

[mpp13435-bib-0090] Kelman, M.J. , Renaud, J.B. , Seifert, K.A. , Mack, J. , Yeung, K.K.‐C. & Sumarah, M.W. (2020) Chemotaxonomic profiling of Canadian *Alternaria* populations using high‐resolution mass spectrometry. Metabolites, 10, 238. 32526912 10.3390/metabo10060238 PMC7345142

[mpp13435-bib-0091] Kępczyńska, E. & Król, P. (2012) The phytohormone methyl jasmonate as an activator of induced resistance against the necrotroph *Alternaria porri* f. sp. *solani* in tomato plants. Journal of Plant Interactions, 7, 307–315.

[mpp13435-bib-0092] Kim, W. , Park, C.‐M. , Park, J.‐J. , Akamatsu, H.O. , Peever, T.L. , Xian, M. et al. (2015) Functional analyses of the diels‐alderase gene *Sol5* of *Ascochyta rabiei* and *Alternaria solani* indicate that the solanapyrone phytotoxins are not required for pathogenicity. Molecular Plant–Microbe Interactions, 28, 482–496. 25372118 10.1094/MPMI-08-14-0234-R

[mpp13435-bib-0093] Kodama, M. (2019) Evolution of pathogenicity in *Alternaria* plant pathogens. Journal of General Plant Pathology, 85, 471–474.

[mpp13435-bib-0094] Kokaeva, L.Y. , Yarmeeva, M.M. , Kokaeva, Z.G. , Chudinova, E.M. , Balabko, P.N. & Elansky, S.N. (2022) Phylogenetic study of *Alternaria* potato and tomato pathogens in Russia. Diversity, 14, 685.

[mpp13435-bib-0095] Kumar, V. , Haldar, S. , Pandey, K.K. , Singh, R.P. , Singh, A.K. & Singh, P.C. (2008) Cultural, morphological, pathogenic and molecular variability amongst tomato isolates of *Alternaria solani* in India. World Journal of Microbiology and Biotechnology, 24, 1003–1009.

[mpp13435-bib-0096] Lagopodi, A.L. & Thanassoulopoulos, C.C. (1995) Development of chlamydospores in *Alternaria alternata* . Mycologia, 87, 588–591.

[mpp13435-bib-0097] Landschoot, S. , Vandecasteele, M. , Carrette, J. , De Baets, B. , Höfte, M. , Audenaert, K. et al. (2017) Assessing the Belgian potato *Alternaria* population for sensitivity to fungicides with diverse modes of action. European Journal of Plant Pathology, 148, 657–672.

[mpp13435-bib-0098] Landschoot, S. , Carrette, J. , Vandecasteele, M. , De Baets, B. , Höfte, M. , Audenaert, K. et al. (2017) Boscalid‐resistance in *Alternaria alternata* and *Alternaria solani* populations: an emerging problem in Europe. Crop Protection, 92, 49–59.

[mpp13435-bib-0099] Landschoot, S. , Vandecasteele, M. , De Baets, B. , Höfte, M. , Audenaert, K. & Haesaert, G. (2017) Identification of *A. arborescens, A. grandis*, and *A. protenta* as new members of the European *Alternaria* population on potato. Fungal Biology, 121, 172–188. 28089048 10.1016/j.funbio.2016.11.005

[mpp13435-bib-0100] Lawrence, D.P. , Gannibal, P.B. , Dugan, F.M. & Pryor, B.M. (2014) Characterization of *Alternaria* isolates from the *infectoria* species‐group and a new taxon from *Arrhenatherum*, *Pseudoalternaria arrhenatheria* sp. nov. Mycological Progress, 13, 257–276.

[mpp13435-bib-0101] Lawrence, D.P. , Gannibal, P.B. , Peever, T.L. & Pryor, B.M. (2013) The sections of *Alternaria*: formalizing species‐group concepts. Mycologia, 105, 530–546. 23687125 10.3852/12-249

[mpp13435-bib-0102] Lawrence, D.P. , Rotondo, F. & Gannibal, P.B. (2015) Biodiversity and taxonomy of the pleomorphic genus *Alternaria* . Mycological Progress, 15, 3.

[mpp13435-bib-0103] Leiminger, J.H. , Adolf, B. & Hausladen, H. (2014) Occurrence of the f 129 l mutation in * alternaria solani* populations in germany in response to qoi application, and its effect on sensitivity. Plant Pathology, 63, 640–650.

[mpp13435-bib-0104] Leiminger, J.H. , Auinger, H.‐J. , Wenig, M. , Bahnweg, G. & Hausladen, H. (2013) Genetic variability among *Alternaria solani* isolates from potatoes in southern Germany based on RAPD‐profiles. Journal of Plant Diseases and Protection, 120, 164–172.

[mpp13435-bib-0105] Leon‐Reyes, A. , Du, Y. , Koornneef, A. , Proietti, S. , Körbes, A.P. , Memelink, J. et al. (2010) Ethylene signaling renders the jasmonate response of *Arabidopsis* insensitive to future suppression by salicylic acid. Molecular Plant–Microbe Interactions, 23, 187–197. 20064062 10.1094/MPMI-23-2-0187

[mpp13435-bib-0106] Li, J.‐F. , Jiang, H.‐B. , Jeewon, R. , Hongsanan, S. , Bhat, D.J. , Tang, S.‐M. et al. (2022) *Alternaria*: update on species limits, evolution, multi‐locus phylogeny, and classification. Studies in Fungi, 8, 1–61.

[mpp13435-bib-0107] Liu, H.F. , Liao, J. , Chen, X.Y. , Liu, Q.K. , Yu, Z.H. & Deng, J.X. (2019) A novel species and a new record of *Alternaria* isolated from two Solanaceae plants in China. Mycological Progress, 18, 1005–1012.

[mpp13435-bib-0108] Lourenço, V., Jr. , Rodrigues, T.T.M.S. , Campos, A.M.D. , Bragança, C.A.D. , Scheuermann, K.K. , Reis, A. et al. (2011) Genetic structure of the population of *Alternaria solani* in Brazil. Journal of Phytopathology, 159, 233–240.

[mpp13435-bib-0109] Lourenço, V. , Moya, A. , González‐Candelas, F. , Carbone, I. , Maffia, L.A. & Mizubuti, E.S.G. (2009) Molecular diversity and evolutionary processes of *Alternaria solani* in Brazil inferred using genealogical and coalescent approaches. Phytopathology, 99, 765–774. 19453237 10.1094/PHYTO-99-6-0765

[mpp13435-bib-0110] Lu, Y. , Xiao, Z. , Xiong, Y. , Yan, J. , Chen, W. , Tang, Q. et al. (2023) Genome sequence resource of *Alternaria alternata* DZ causing tobacco brown spot. PhytoFrontiers, 3, 898–901.

[mpp13435-bib-0111] Mallik, I. , Arabiat, S. , Pasche, J.S. , Bolton, M.D. , Patel, J.S. & Gudmestad, N.C. (2013) Molecular characterization and detection of mutations associated with resistance to succinate dehydrogenase‐inhibiting fungicides in *Alternaria solani* . Phytopathology, 104, 40–49. 10.1094/PHYTO-02-13-0041-R 23901829

[mpp13435-bib-0112] Mang, H.G. , Laluk, K.A. , Parsons, E.P. , Kosma, D.K. , Cooper, B.R. , Park, H.C. et al. (2009) The *Arabidopsis* RESURRECTION1 gene regulates a novel antagonistic interaction in plant defense to biotrophs and necrotrophs. Plant Physiology, 151, 290–305. 19625635 10.1104/pp.109.142158 PMC2735982

[mpp13435-bib-0113] Masunaka, A. , Ohtani, K. , Peever, T.L. , Timmer, L.W. , Tsuge, T. , Yamamoto, M. et al. (2005) An isolate of *Alternaria alternata* that is pathogenic to both tangerines and rough lemon and produces two host‐selective toxins, ACT‐ and ACR‐toxins. Phytopathology, 95, 241–247. 18943116 10.1094/PHYTO-95-0241

[mpp13435-bib-0114] Meena, M. , Gupta, S.K. , Swapnil, P. , Zehra, A. , Dubey, M.K. & Upadhyay, R.S. (2017) *Alternaria* toxins: potential virulence factors and genes related to pathogenesis. Frontiers in Microbiology, 8, 1451. 28848500 10.3389/fmicb.2017.01451 PMC5550700

[mpp13435-bib-0115] Meena, M. , Zehra, A. , Dubey, M.K. , Aamir, M. , Gupta, V.K. & Upadhyay, R.S. (2016) Comparative evaluation of biochemical changes in tomato (*Lycopersicon esculentum* Mill.) infected by *Alternaria alternata* and its toxic metabolites (TeA, AOH, and AME). Frontiers in Plant Science, 7, 1408. 27713751 10.3389/fpls.2016.01408 PMC5031594

[mpp13435-bib-0116] Mehrabi, R. , Bahkali, A.H. , Abd‐Elsalam, K.A. , Moslem, M. , Ben M'Barek, S. , Gohari, A.M. et al. (2011) Horizontal gene and chromosome transfer in plant pathogenic fungi affecting host range. FEMS Microbiology Reviews, 35, 542–554. 21223323 10.1111/j.1574-6976.2010.00263.x

[mpp13435-bib-0117] Meng, D. , Li, C. , Park, H.‐J. , González, J. , Wang, J. , Dandekar, A.M. et al. (2018) Sorbitol modulates resistance to *Alternaria alternata* by regulating the expression of an NLR resistance gene in apple. The Plant Cell, 30, 1562–1581. 29871985 10.1105/tpc.18.00231 PMC6096587

[mpp13435-bib-0118] Meng, J.‐W. , Zhu, W. , He, M.‐H. , Wu, E.‐J. , Duan, G.‐H. , Xie, Y.‐K. et al. (2015) Population genetic analysis reveals cryptic sex in the phytopathogenic fungus *Alternaria* alternata. Scientific Reports, 5, 18250. 26666175 10.1038/srep18250 PMC4678894

[mpp13435-bib-0119] Mengiste, T. (2012) Plant immunity to necrotrophs. Annual Review of Phytopathology, 50, 267–294. 10.1146/annurev-phyto-081211-172955 22726121

[mpp13435-bib-0120] Morris, P.F. , Connolly, M.S. & St Clair, D.A. (2000) Genetic diversity of *Alternaria alternata* isolated from tomato in California assessed using RAPDs. Mycological Research, 104, 286–292.

[mpp13435-bib-0121] Nakayasu, M. , Shioya, N. , Shikata, M. , Thagun, C. , Abdelkareem, A. , Okabe, Y. et al. (2018) JRE4 is a master transcriptional regulator of defense‐related steroidal glycoalkaloids in tomato. The Plant Journal, 94, 975–990. 29569783 10.1111/tpj.13911

[mpp13435-bib-0122] Nasr‐Esfahani, M. , Gholamaliyan, R. , Esmaiili, A. & Mardani, S. (2021) Genomic structure and population genetic analysis of leaf spot disease, *Ulocladium atrum* ‐potato pathotype isolates. Acta Horticulturae, 1326, 255–264.

[mpp13435-bib-0123] Nishikawa, J. & Nakashima, C. (2020) Japanese species of *Alternaria* and their species boundaries based on host range. Fungal Systematics and Evolution, 5, 197–282. 32467924 10.3114/fuse.2020.05.13 PMC7250166

[mpp13435-bib-0124] Nottensteiner, M. , Absmeier, C. & Zellner, M. (2019) QoI fungicide resistance mutations in *Alternaria solani* and *Alternaria alternata* are fully established in potato growing areas in Bavaria and dual resistance against SDHI fungicides is upcoming. Gesunde Pflanzen, 71, 155–164.

[mpp13435-bib-0125] Odilbekov, F. , Selga, C. , Ortiz, R. , Chawade, A. & Liljeroth, E. (2020) QTL mapping for resistance to early blight in a tetraploid potato population. Agronomy, 10, 728.

[mpp13435-bib-0126] Orina, A.S. , Gannibal, P.H.B. & Levitin, M.M. (2010) Specific diversity, biological characters and geography of *Alternaria* fungi associated with solanaceous plants. Mikologiya i Fitopatologiya, 44, 150–159.

[mpp13435-bib-0127] Osbourn, A. (1996) Saponins and plant defence—a soap story. Trends in Plant Science, 1, 4–9.

[mpp13435-bib-0128] Ozkilinc, H. , Rotondo, F. , Pryor, B.M. & Peever, T.L. (2018) Contrasting species boundaries between sections *Alternaria* and *Porri* of the genus *Alternaria* . Plant Pathology, 67, 303–314.

[mpp13435-bib-0129] Pasche, J.S. & Gudmestad, N.C. (2008) Prevalence, competitive fitness and impact of the F129L mutation in *Alternaria solani* from the United States. Crop Protection, 27, 427–435.

[mpp13435-bib-0130] Pasche, J.S. , Wharam, C.M. & Gudmestad, N.C. (2004) Shift in sensitivity of *Alternaria solani* in response to QoI fungicides. Plant Disease, 88, 181–187. 30812426 10.1094/PDIS.2004.88.2.181

[mpp13435-bib-0131] Patterson, C.L. (1991) Importance of chlamydospores as primary inoculum for *Alternaria solani*, incitant of collar rot and early blight on tomato. Plant Disease, 75, 274.

[mpp13435-bib-0132] Peixoto, C.C. , Cabral, C.S. , Fonseca, M.E.N. , Boiteux, L.S. & Reis, A. (2021) Species diversity, novel interactions and absence of well‐supported host‐guided phylogenetic groupings of neotropical *Alternaria* isolates causing foliar lesions in Solanaceae. Journal of Applied Microbiology, 131, 2466–2487. 33891782 10.1111/jam.15115

[mpp13435-bib-0133] Petrunak, D.M. & Christ, B.J. (1992) Isozyme variability in *Alternaria solani* and *A. alternata* . Phytopathology, 82, 1343.

[mpp13435-bib-0134] Pinto, V.E.F. & Patriarca, A. (2017) *Alternaria* species and their associated mycotoxins. In: Moretti, A. & Susca, A. (Eds.) Mycotoxigenic fungi: methods and protocols. Methods in molecular biology. New York, NY: Springer, pp. 13–32. 10.1007/978-1-4939-6707-0_2 27924529

[mpp13435-bib-0135] Prakash, N. , Vishunavat, K. & Prasad, P. (2022) Diversity analysis of *Alternaria solani* isolates infecting tomato in Uttarakhand, India. Indian Phytopathology, 75, 331–343.

[mpp13435-bib-0136] Puntscher, H. , Marko, D. & Warth, B. (2019) The fate of altertoxin II during tomato processing steps at a laboratory scale. Frontiers in Nutrition, 6, 92. 31263702 10.3389/fnut.2019.00092 PMC6584911

[mpp13435-bib-0137] Ramezani, Y. , Taheri, P. & Mamarabadi, M. (2019) Identification of *Alternaria* spp. associated with tomato early blight in Iran and investigating some of their virulence factors. Journal of Plant Pathology, 101, 647–659.

[mpp13435-bib-0138] Rich, A.E. (2013) Potato diseases. New York: Academic Press.

[mpp13435-bib-0139] Roberts, R.G. , Bischoff, J.F. & Reymond, S.T. (2012) Differential gene expression in *Alternaria gaisen* exposed to dark and light. Mycological Progress, 11, 373–382.

[mpp13435-bib-0140] Roberts, R.G. , Reymond, S.T. & Andersen, B. (2000) RAPD fragment pattern analysis and morphological segregation of small‐spored *Alternaria* species and species groups. Mycological Research, 104, 151–160.

[mpp13435-bib-0141] Robert‐Seilaniantz, A. , Grant, M. & Jones, J.D.G. (2011) Hormone crosstalk in plant disease and defense: more than just jasmonate‐salicylate antagonism. Annual Review of Phytopathology, 49, 317–343. 10.1146/annurev-phyto-073009-114447 21663438

[mpp13435-bib-0142] Rodrigues, T.T.M.S. , Berbee, M.L. , Simmons, E.G. , Cardoso, C.R. , Reis, A. , Maffia, L.A. et al. (2010) First report of *Alternaria tomatophila* and *A. grandis* causing early blight on tomato and potato in Brazil. New Disease Reports, 22, 28.

[mpp13435-bib-0143] Rotem, J. (1994) The genus Alternaria: biology, epidemiology, and pathogenicity. St. Paul, MN: APS Press.

[mpp13435-bib-0144] Sadeghi, B. , Mirzaei, S. & Fatehi, F. (2022) The proteomic analysis of the resistance responses in tomato during interaction with *Alternaria alternata* . Scientia Horticulturae, 304, 111295.

[mpp13435-bib-0145] Sajeevan, R.S. , Abdelmeguid, I. , Saripella, G.V. , Lenman, M. & Alexandersson, E. (2023) Comprehensive transcriptome analysis of different potato cultivars provides insight into early blight disease caused by *Alternaria solani* . BMC Plant Biology, 23, 130. 36882678 10.1186/s12870-023-04135-9 PMC9993742

[mpp13435-bib-0146] Sandrock, R.W. & VanEtten, H.D. (1998) Fungal sensitivity to and enzymatic degradation of the phytoanticipin α‐tomatine. Phytopathology, 88, 137–143. 18944982 10.1094/PHYTO.1998.88.2.137

[mpp13435-bib-0147] Schmey, T. , Small, C. , Einspanier, S. , Hoyoz, L.M. , Ali, T. , Gamboa, S. et al. (2023) Small‐spored *Alternaria* spp. (section *Alternaria*) are common pathogens on wild tomato species. Environmental Microbiology, 25, 1830–1846. 37171093 10.1111/1462-2920.16394

[mpp13435-bib-0148] Schoch, C.L. , Seifert, K.A. , Huhndorf, S. , Robert, V. , Spouge, J.L. , Levesque, C.A. et al. (2012) Nuclear ribosomal internal transcribed spacer (ITS) region as a universal DNA barcode marker for fungi. Proceedings of the National Academy of Sciences of the United States of America, 109, 6241–6246. 22454494 10.1073/pnas.1117018109 PMC3341068

[mpp13435-bib-0149] Schweizer, P. , Felix, G. , Buchala, A. , Müller, C. & Métraux, J.‐P. (1996) Perception of free cutin monomers by plant cells. The Plant Journal, 10, 331–341.

[mpp13435-bib-0150] Shahbazi, H. , Aminian, H. , Sahebani, N. & Halterman, D. (2011) Effect of *Alternaria solani* exudates on resistant and susceptible potato cultivars from two different pathogen isolates. The Plant Pathology Journal, 27, 14–19.

[mpp13435-bib-0151] Shinde, B.A. , Dholakia, B.B. , Hussain, K. , Panda, S. , Meir, S. , Rogachev, I. et al. (2017) Dynamic metabolic reprogramming of steroidal glycol‐alkaloid and phenylpropanoid biosynthesis may impart early blight resistance in wild tomato (*Solanum arcanum* Peralta). Plant Molecular Biology, 95, 411–423. 28980117 10.1007/s11103-017-0660-2

[mpp13435-bib-0152] Simmons, E.G. (2000) *Alternaria* themes and variations (244‐286) species on Solanaceae. Mycotaxon, 75, 1–115.

[mpp13435-bib-0153] Somma, S. , Pose, G. , Pardo, A. , Mulè, G. , Pinto, V.F. , Moretti, A. et al. (2011) AFLP variability, toxin production, and pathogenicity of *Alternaria* species from Argentinean tomato fruits and puree. International Journal of Food Microbiology, 145, 414–419. 21303723 10.1016/j.ijfoodmicro.2011.01.006

[mpp13435-bib-0154] Soukup, S.T. , Kohn, B.N. , Pfeiffer, E. , Geisen, R. , Metzler, M. , Bunzel, M. et al. (2016) Sulfoglucosides as novel modified forms of the mycotoxins alternariol and alternariol monomethyl ether. Journal of Agricultural and Food Chemistry, 64, 8892–8901. 27776211 10.1021/acs.jafc.6b03120

[mpp13435-bib-0155] Stack, M.E. & Prival, M.J. (1986) Mutagenicity of the *Alternaria* metabolites altertoxins I, II, and III. Applied and Environmental Microbiology, 52, 718–722. 3535674 10.1128/aem.52.4.718-722.1986 PMC239103

[mpp13435-bib-0156] Stall, R.E. (1958) An investigation of nuclear number in *Alternaria solani* . American Journal of Botany, 45, 657–659.

[mpp13435-bib-0157] Stewart, J.E. , Andrew, M. , Bao, X. , Chilvers, M.I. , Carris, L.M. & Peever, T.L. (2013) Development of sequence characterized amplified genomic regions (SCAR) for fungal systematics: proof of principle using *Alternaria*, *Ascochyta* and *Tilletia* . Mycologia, 105, 1077–1086. 23449078 10.3852/12-287

[mpp13435-bib-0158] Stewart, J.E. , Kawabe, M. , Abdo, Z. , Arie, T. & Peever, T.L. (2011) Contrasting codon usage patterns and purifying selection at the mating locus in putatively asexual *Alternaria* fungal species. PLoS One, 6, e20083. 21625561 10.1371/journal.pone.0020083 PMC3098265

[mpp13435-bib-0159] Stewart, J.E. , Thomas, K.A. , Lawrence, C.B. , Dang, H. , Pryor, B.M. , Timmer, L.M. et al. (2013) Signatures of recombination in clonal lineages of the citrus brown spot pathogen, *Alternaria alternata* sensu lato. Phytopathology, 103, 741–749. 23441968 10.1094/PHYTO-08-12-0211-R

[mpp13435-bib-0160] Stewart, J.E. , Timmer, L.W. , Lawrence, C.B. , Pryor, B.M. & Peever, T.L. (2014) Discord between morphological and phylogenetic species boundaries: incomplete lineage sorting and recombination results in fuzzy species boundaries in an asexual fungal pathogen. BMC Evolutionary Biology, 14, 38. 24593138 10.1186/1471-2148-14-38 PMC4015827

[mpp13435-bib-0161] Suemitsu, R. , Yamada, Y. , Sano, T. & Yamashita, K. (1984) Phytotoxic activities of altersolanol A, B and dactylariol, and activities of altersolanol A against some microorganisms. Agricultural and Biological Chemistry, 48, 2383–2384.

[mpp13435-bib-0162] Sun, C. , Li, F. , Wei, M. , Xiang, Z. , Chen, C. & Xu, D. (2021) Detection and biological characteristics of *Alternaria alternata* resistant to difenoconazole from *Paris polyphylla* var. *chinensis*, an indigenous medicinal herb. Plant Disease, 105, 1546–1554. 33349004 10.1094/PDIS-12-19-2699-RE

[mpp13435-bib-0163] Tanabe, K. , Tsuge, T. & Nishimura, S. (1989) Potential application of DNA restriction fragment length polymorphisms to the ecological studies of *Alternaria alternata* Japanese pear pathotype. Japanese Journal of Phytopathology, 55, 361–365.

[mpp13435-bib-0164] Tanaka, A. & Tsuge, T. (2000) Structural and functional complexity of the genomic region controlling AK‐toxin biosynthesis and pathogenicity in the Japanese pear pathotype of *Alternaria alternata* . Molecular Plant–Microbe Interactions, 13, 975–986. 10975654 10.1094/MPMI.2000.13.9.975

[mpp13435-bib-0165] Taylor, J.W. , Hann‐Soden, C. , Branco, S. , Sylvain, I. & Ellison, C.E. (2015) Clonal reproduction in fungi. Proceedings of the National Academy of Sciences of the United States of America, 112, 8901–8908. 26195774 10.1073/pnas.1503159112 PMC4517272

[mpp13435-bib-0166] Thaler, J.S. , Humphrey, P.T. & Whiteman, N.K. (2012) Evolution of jasmonate and salicylate signal crosstalk. Trends in Plant Science, 17, 260–270. 22498450 10.1016/j.tplants.2012.02.010

[mpp13435-bib-0167] Thomidis, T. , Prodromou, I. , Paresidou, M. & Damos, P. (2023) Effects of temperature and leaf wetness duration on pathogens causing preharvest fruit rots on tomato. Journal of Plant Pathology, 105, 1431–1448.

[mpp13435-bib-0168] Thomma, B.P.H.J. (2003) *Alternaria* spp.: from general saprophyte to specific parasite. Molecular Plant Pathology, 4, 225–236. 20569383 10.1046/j.1364-3703.2003.00173.x

[mpp13435-bib-0169] Tominello‐Ramirez, C. , Hoyos, L.M. , Oubounyt, M. & Stam, R. (2023) Network analyses reveal D clade ethylene response factors as major regulators of jasmonic acid‐mediated resistance to early blight disease complex in tomato. *bioRxiv* [Preprint] doi: 10.1101/2023.10.14.562343

[mpp13435-bib-0170] Tsuge, T. , Harimoto, Y. , Akimitsu, K. , Ohtani, K. , Kodama, M. , Akagi, Y. et al. (2013) Host‐selective toxins produced by the plant pathogenic fungus *Alternaria alternata* . FEMS Microbiology Reviews, 37, 44–66. 22846083 10.1111/j.1574-6976.2012.00350.x

[mpp13435-bib-0171] Tsuge, T. , Harimoto, Y. , Hanada, K. , Akagi, Y. , Kodama, M. , Akimitsu, K. et al. (2016) Evolution of pathogenicity controlled by small, dispensable chromosomes in *Alternaria alternata* pathogens. Physiological and Molecular Plant Pathology, 95, 27–31.

[mpp13435-bib-0172] Tsuge, T. , Hayashi, N. & Nishimura, S. (1987) [Selection of auxotrophic mutants and heterokaryosis in *Alternaria alternata* .] Nippon Shokubutsu Byori Gakkaiho, 53, 182–190.

[mpp13435-bib-0173] Tymon, L.S. , Cummings, T.F. & Johnson, D.A. (2016) Pathogenicity and aggressiveness of three *Alternaria* spp. on potato foliage in the U.S. Northwest. Plant Disease, 100, 797–801. 30688619 10.1094/PDIS-08-15-0942-RE

[mpp13435-bib-0174] Tymon, L.S. , Peever, T.L. & Johnson, D.A. (2016) Identification and enumeration of small‐spored *Alternaria* species associated with potato in the U.S. Northwest. Plant Disease, 100, 465–472. 30694153 10.1094/PDIS-03-15-0263-RE

[mpp13435-bib-0175] Upadhyay, P. , Ganaie, S.H. & Singh, N. (2019) Diversity assessment among *Alternaria solani* isolates causing early blight of tomato in India. Proceedings of the National Academy of Sciences, India Section B: Biological Sciences, 89, 987–997.

[mpp13435-bib-0176] van der Waals, J.E. , Korsten, L. & Slippers, B. (2004) Genetic diversity among *Alternaria solani* isolates from potatoes in South Africa. Plant Disease, 88, 959–964. 30812248 10.1094/PDIS.2004.88.9.959

[mpp13435-bib-0178] van Wyk, S. , Wingfield, B.D. , De Vos, L. , Merwe, N.A.v.d. & Steenkamp, E.T. (2021) Genome‐wide analyses of repeat‐induced point mutations in the Ascomycota. Frontiers in Microbiology, 11, 622368. 33597932 10.3389/fmicb.2020.622368 PMC7882544

[mpp13435-bib-0179] Vandecasteele, M. , Landschoot, S. , Carrette, J. , Verwaeren, J. , Höfte, M. , Audenaert, K. et al. (2018) Species prevalence and disease progression studies demonstrate a seasonal shift in the *Alternaria* population composition on potato. Plant Pathology, 67, 327–336.

[mpp13435-bib-0211] VanEtten, H.D. , Mansfield, J.W. , Bailey, J.A. & Farmer, E.E. (1994) Two classes of plant antibiotics: phytoalexins versus “phytoanticipins”. The Plant Cell, 6, 1191–1192. 12244269 10.1105/tpc.6.9.1191 PMC160512

[mpp13435-bib-0180] Varma, P.K. , Singh, S. & Gandhi, S.K. (2007) Variability among *Alternaria solani* isolates causing early blight of tomato. Indian Phytopathology, 60, 180. 17612350

[mpp13435-bib-0181] Visconti, A. , Bottalico, A. & Solfrizzo, M. (1989) Activity of *Alternaria alternata* metabolites on tomato leaves and *Geotrichum candidum* . In: Graniti, A. , Durbin, R.D. & Ballio, A. (Eds.) Phytotoxins and plant pathogenesis NATO ASI series, Vol. 27. Berlin, Heidelberg: Springer, pp. 457–459.

[mpp13435-bib-0182] Wang, C. , Wang, J. , Zhang, D. , Cheng, J. , Zhu, J. & Yang, Z. (2023) Identification and functional analysis of protein secreted by *Alternaria solani* . PLoS One, 18, e0281530. 36877688 10.1371/journal.pone.0281530 PMC9987770

[mpp13435-bib-0183] Wang, C. , Zhang, D. , Cheng, J. , Zhao, D. , Pan, Y. , Li, Q. et al. (2022) Identification of effector CEP112 that promotes the infection of necrotrophic *Alternaria solani* . BMC Plant Biology, 22, 466. 36171557 10.1186/s12870-022-03845-w PMC9520946

[mpp13435-bib-0184] Wang, J. , Xiao, S. , Zheng, L. , Pan, Y. , Zhao, D. , Zhang, D. et al. (2022) Multiomic approaches reveal novel lineage‐specific effectors in the potato and tomato early blight pathogen *Alternaria solani* . Phytopathology Research, 4, 29.

[mpp13435-bib-0185] Wang, M. , Fu, H. & Ruan, R. (2019) A small horizontally transferred gene cluster contributes to the sporulation of *Alternaria alternata* . Genome Biology and Evolution, 11, 3436–3444. 31764979 10.1093/gbe/evz257 PMC6916707

[mpp13435-bib-0186] Wang, Y. , Pei, Y.‐F. , O'Neill, N.R. & Zhang, X.‐G. (2010) *Ulocladium cantlous* sp. nov. isolated from northwestern China: its morphology and molecular phylogenetic position. Mycologia, 102, 374–383. 20361504 10.3852/09-093

[mpp13435-bib-0187] Wang, Y. , Pruitt, R.N. , Nürnberger, T. & Wang, Y. (2022) Evasion of plant immunity by microbial pathogens. Nature Reviews Microbiology, 20, 449–464. 35296800 10.1038/s41579-022-00710-3

[mpp13435-bib-0188] Weber, B. & Halterman, D.A. (2012) Analysis of genetic and pathogenic variation of *Alternaria solani* from a potato production region. European Journal of Plant Pathology, 134, 847–858.

[mpp13435-bib-0189] Weir, T.L. , Huff, D.R. , Christ, B.J. & Romaine, C.P. (1998) RAPD‐PCR analysis of genetic variation among isolates of *Alternaria solani* and *Alternaria alternata* from potato and tomato. Mycologia, 90, 813–821.

[mpp13435-bib-0190] Wenderoth, M. , Garganese, F. , Schmidt‐Heydt, M. , Soukup, S.T. , Ippolito, A. , Sanzani, S.M. et al. (2019) Alternariol as virulence and colonization factor of *Alternaria alternata* during plant infection. Molecular Microbiology, 112, 131–146. 30947377 10.1111/mmi.14258

[mpp13435-bib-0191] Wharton, P. , Fairchild, K. , Belcher, A. & Wood, E. (2012) First report of in‐vitro boscalid‐resistant isolates of *Alternaria solani* causing early blight of potato in Idaho. Plant Disease, 96, 454. 10.1094/PDIS-07-11-0544 30727105

[mpp13435-bib-0192] Witsenboer, H.M.A. , Kloosterziel, K.M. , Hateboer, G. , Nijkamp, H.J.J. & Hille, J. (1992) Tomato susceptibility to *Alternaria* stem canker: parameters involved in host‐specific toxin‐induced leaf necrosis. Plant Science, 81, 127–134.

[mpp13435-bib-0193] Wolters, P.J. , Faino, L. , Bosch, T.B.M.v.d. , Evenhuis, B. , Visser, R.G.F. , Seidl, M.F. et al. (2018) Gapless genome assembly of the potato and tomato early blight pathogen *Alternaria solani* . Molecular Plant–Microbe Interactions, 31, 692–694. 29432053 10.1094/MPMI-12-17-0309-A

[mpp13435-bib-0194] Wolters, P.J. , Vos, L.d. , Bijsterbosch, G. , Woudenberg, J.H.C. , Visser, R.G.F. , Linden, G.v.d. et al. (2019) A rapid method to screen wild *Solanum* for resistance to early blight. European Journal of Plant Pathology, 154, 109–114.

[mpp13435-bib-0195] Wolters, P.J. , Wouters, D. , Kromhout, E.J. , Huigen, D.J. , Visser, R.G.F. & Vleeshouwers, V.G.A.A. (2021) Qualitative and quantitative resistance against early blight introgressed in potato. Biology, 10, 892. 34571769 10.3390/biology10090892 PMC8471710

[mpp13435-bib-0196] Wolters, P.J. , Wouters, D. , Tikunov, Y.M. , Ayilalath, S. , Kodde, L.P. , Strijker, M.F. et al. (2023) Tetraose steroidal glycoalkaloids from potato provide resistance against *Alternaria solani* and Colorado potato beetle. eLife, 12, RP87135. 37751372 10.7554/eLife.87135 PMC10522338

[mpp13435-bib-0197] Woudenberg, J.H.C. , Groenewald, J.Z. , Binder, M. & Crous, P.W. (2013) *Alternaria* redefined. Studies in Mycology, 75, 171–212. 24014900 10.3114/sim0015 PMC3713888

[mpp13435-bib-0198] Woudenberg, J.H.C. , Seidl, M.F. , Groenewald, J.Z. , de Vries, M. , Stielow, J.B. , Thomma, B.P.H.J. et al. (2015) *Alternaria* section *Alternaria*: species, formae speciales or pathotypes? Studies in Mycology, 82, 1–21. 26951037 10.1016/j.simyco.2015.07.001 PMC4774270

[mpp13435-bib-0199] Woudenberg, J.H.C. , Truter, M. , Groenewald, J.Z. & Crous, P.W. (2014) Large‐spored *Alternaria* pathogens in section *Porri* disentangled. Studies in Mycology, 79, 1–47. 25492985 10.1016/j.simyco.2014.07.003 PMC4255562

[mpp13435-bib-0200] Xue, W. , Haynes, K.G. , Clarke, C.R. & Qu, X. (2022) Genetic dissection of early blight resistance in tetraploid potato. Frontiers in Plant Science, 13, 851538. 35401646 10.3389/fpls.2022.851538 PMC8990756

[mpp13435-bib-0201] Zachmann, R. (1982) Early blight of potato Alternaria solani. Technical information bulletin 17. Lima: International Potato Center.

[mpp13435-bib-0202] Zhang, D. , He, J.‐Y. , Haddadi, P. , Zhu, J.‐H. , Yang, Z.‐H. & Ma, L. (2018) Genome sequence of the potato pathogenic fungus *Alternaria solani* HWC‐168 reveals clues for its conidiation and virulence. BMC Microbiology, 18, 176. 30400851 10.1186/s12866-018-1324-3 PMC6219093

[mpp13435-bib-0203] Zhang, L. , Jia, C. , Liu, L. , Zhang, Z. , Li, C. & Wang, Q. (2011) The involvement of jasmonates and ethylene in *Alternaria alternata* f. sp. *lycopersici* toxin‐induced tomato cell death. Journal of Experimental Botany, 62, 5405–5418. 21865178 10.1093/jxb/err217 PMC3223041

[mpp13435-bib-0204] Zhang, Y. , Zhou, Q. , Tian, P. , Li, Y. , Duan, G. , Li, D. et al. (2020) Induced expression of *CYP51* associated with difenoconazole resistance in the pathogenic *Alternaria* sect. on potato in China. Pest Management Science, 76, 1751–1760. 31785067 10.1002/ps.5699

[mpp13435-bib-0205] Zhao, D. , Fan, S. , Zhang, D. , Pan, Y. , Gu, Q. , Wang, J. et al. (2021) Parasexual reproduction in *Alternaria solani*: simple sequence repeat molecular evidence for haploidization. Mycologia, 113, 949–955. 34125655 10.1080/00275514.2021.1922243

[mpp13435-bib-0206] Zhao, D.‐K. , Zhao, Y. , Chen, S.‐Y. & Kennelly, E.J. (2021) *Solanum* steroidal glycoalkaloids: structural diversity, biological activities, and biosynthesis. Natural Product Reports, 38, 1423–1444. 35226001 10.1039/d1np00001b

[mpp13435-bib-0207] Zhao, L. , Cheng, H. , Liu, H.‐F. , Gao, G.‐Y. , Zhang, Y. , Li, Z.‐N. et al. (2023) Pathogenicity and diversity of large‐spored *Alternaria* associated with three solanaceous vegetables (*Solanum tuberosum, S. lycopersicum* and *S. melongena*) in China. Plant Pathology, 72, 376–391.

[mpp13435-bib-0208] Zheng, L. , Yang, P. , Niu, Z. , Tian, M. , Wang, J. , Sun, C. et al. (2022) Dissecting in vivo responses of phytohormones to *Alternaria solani* infection reveals orchestration of JA‐ and ABA‐mediated antifungal defenses in potato. Horticulture Research, 9, uhac188. 37180032 10.1093/hr/uhac188 PMC10167417

[mpp13435-bib-0209] Zhou, B. , Wang, H. , Meng, B. , Wei, R. , Wang, L. , An, C. et al. (2019) An evaluation of tenuazonic acid, a potential biobased herbicide in cotton. Pest Management Science, 75, 2482–2489. 30843361 10.1002/ps.5402

[mpp13435-bib-0210] Zwickel, T. , Kahl, S.M. , Rychlik, M. & Müller, M.E.H. (2018) Chemotaxonomy of mycotoxigenic small‐spored *Alternaria* fungi—do multitoxin mixtures act as an indicator for species differentiation? Frontiers in Microbiology, 9, 1368. 30018598 10.3389/fmicb.2018.01368 PMC6037717

